# Transcriptome analysis of *Homo sapiens* and *Mus musculus* reveals mechanisms of CD8+ T cell exhaustion caused by different factors

**DOI:** 10.1371/journal.pone.0274494

**Published:** 2022-09-09

**Authors:** Lin Zhang, Hafumi Nishi

**Affiliations:** 1 Graduate School of Information Sciences, Tohoku University, Sendai, Japan; 2 Faculty of Core Research, Ochanomizu University, Tokyo, Japan; 3 Tohoku Medical Megabank Organization, Tohoku University, Sendai, Japan; University of Pittsburgh, UNITED STATES

## Abstract

T cell exhaustion is a state of T cell dysfunction during chronic infection and cancer. Antibody-targeting immune checkpoint inhibitors to reverse T cell exhaustion is a promising approach for cancer immunotherapy. However, molecular mechanisms of T cell exhaustion remain incompletely understood. Here, we performed a transcriptome analysis by integrating seven exhaustion datasets caused by multiple diseases in both humans and mice. In this study, an overlap of 21 upregulated and 37 downregulated genes was identified in human and mouse exhausted CD8+ T cells. These genes were significantly enriched in exhaustion response-related pathways, such as signal transduction, immune system processes, and regulation of cytokine production. Gene expression network analysis revealed that the well-documented exhaustion genes were defined as hub genes in upregulated genes. In addition, a weighted gene co-expression analysis identified 175 overlapping genes that were significantly correlated with the exhaustion trait in both humans and mice. This study found that overlapping six genes were significantly upregulated and highly related to T cell exhaustion. Finally, we revealed that CD200R1 and ADGRG1, less described previously in exhaustion, contributed to T cell exhaustion. Overall, our findings reveal the mechanisms of T cell exhaustion and provide an important reference to the immunology community.

## Introduction

T cell exhaustion, a state of differentiation that may develop independently from normal memory and effector T cells during chronic infections, sepsis, and cancer, is antigen dependent and usually exhibits both poor effector functions in a hierarchical manner and impaired memory T cell potential [1–4]. Typically, (i) the robust proliferative and multiple cytokine production capacities that include interferon-γ (IFNγ), tumor necrosis factor (TNF), and interleukin-2 (IL-2) are lost in exhausted cells; (ii) inhibitory checkpoint receptors are expressed in increasing amounts and diversity, such as programmed cell death protein 1 (PD1), lymphocyte activation gene 3 protein (LAG3), hepatitis A virus cellular receptor 2 (HAVCR2/TIM3), cytotoxic T lymphocyte antigen 4 (CTLA4), CD160, 2B4, B- and T-lymphocyte attenuator (BTLA), and T cell immunoreceptor with immunoglobulin and ITIM domains (TIGIT); and (iii) changes in transcriptional profiles and metabolic derangements [2, 3, 5–8]. Exhausted T cells maintain their functional exhaustion after exposure to antigens [9]. They were initially identified in chronic viral infections in mice [10, 11], and similar dysfunctional features have been observed in cancer [12–14].

Intriguingly, although exhausted T cells are often related to inefficient clearance of persistent infections and tumors, immune responses may be reinvigorated in exhausted T cells by targeting inhibitory checkpoint receptors [2]. For example, the function of exhausted CD8+ T cells present in mice during persistent lymphocytic choriomeningitis virus (LCMV) infection is restored by blocking the interaction of PD1 with its ligand PD-L1 [15]. Likewise, blockade of the inhibitory receptor CTLA4 allows transient reprogramming and functional rescue of T cell dysfunction due to the tumor microenvironment [12]. Of note, antibody blockade of immune checkpoints is considered a promising approach for cancer immunotherapy and therapeutic intervention of virus- or tumor-specific T cells during chronic viral infections or tumors [12, 16, 17]. Fortunately, certain antibodies have been developed with good clinical performance; for example, the antibody ipilimumab, targeting the CTLA4 pathway, has achieved US Food and Drug Administration approval as the first immunotherapy for the treatment of melanoma [17]. Furthermore, PD-1 inhibitors nivolumab and pembrolizumab have been approved and win the 2018 Nobel Prize [18, 19]. First LAG-3-blocking antibody combination, Opdualag (nivolumab and relatlimab-rmbw), has also recently been approved as treatment for patients with unresectable or metastatic melanoma on March 18, 2022 [20]. Checkpoint blockade has revolutionized how cancer is treated. However, it is important to emphasize that immunotherapeutics remain problematic. For example, exhausted T cells manifest heterogeneous populations, such as T-bet^hi^ PD1^mid^ and eomesodermin (EOMES)^hi^ PD1^hi^ subsets, but only the T-bet^hi^ PD1^mid^ subset, rather than EOMES^hi^ PD1^hi^, is reinvigorated by blockade of the PD1 pathway [21, 22]. In addition, the effectiveness of the PD1 pathway blockade depends on the pre-existing antitumor immune response in patients. Generally, targeting both PD1 and the CTLA4 pathway leads to adverse immune-related toxicities in patients [23], but it has been reported that this varies from individual to individual [24].

Therefore, here, virus- and tumor-specific exhausted CD8+ T cells were investigated to uncover the molecular mechanisms of CD8+ T cell dysfunction and also provide an important reference for therapeutic blockade in the treatment of exhausted T cells. Of particular note is that in mice models with cancer, antitumor immunity is enhanced via blocking multiple immune checkpoints [16, 25]; thus, it is possible to make large achievements in immunotherapeutics if more targets are found. In this study, raw RNA-sequencing (RNA-seq) read datasets were collected from human and mouse samples, representing either normal or exhausted T cells. Eventually, we revealed that the CD200R1 and ADGRG1 played a vital role in understanding T cell dysfunction caused by chronic infections and other diseases.

## Materials and methods

### Acquisition of the RNA-seq datasets

Raw RNA-seq reads of CD8+ T cells were collected from Gene expression omnibus (GEO). Three datasets were from *Homo sapiens* studies [26–28], and four datasets were from *Mus musculus* [29–32]. Regarding the datasets, CD8+ T cell exhaustion occurs during non-small-cell lung cancer, hepatocellular carcinoma, type 1 diabetes (T1D), or chronic infection. Samples were further filtered using the following criteria: (i) CD8+ T cell samples were used; (ii) transgenic and PD1 blockade samples were excluded; (iii) in the T1D study, T cell immunoreceptor with Ig and ITIM domains (TIGIT)^+^ killer cell lectin like receptor G1 (KLRG1)^+^ [double high (DH)] and TIGIT^-^KLRG1^-^ [double low (DL)] CD8+ T cells were used after 6 months of teplizumab antibody treatment, since the authors investigated that DH cells exhibited an exhaustion phenotype compared to DL cells. Moreover, DH cells treated with poliovirus receptor-Fc (a ligand for TIGIT) were excluded because they were downregulated. The accession numbers reported in this paper were GSE99531, GSE111389, GSE85530, GSE93006, GSE84820, GSE83978, and GSE86881.

### RNA-seq pipeline analysis

The raw RNA-seq reads were filtered using the FASTX-Toolkit fastq_quality_filter [33] by setting the parameters -q 20 (minimum quality score to keep) and -p 80 (minimum percent of bases that must have [-q] quality). Duplications caused by polymerase chain reaction amplification were removed such that paired- and single-end reads used fastuniq [33] and the clumpify.sh program from BBMap suite v38.32 [34], respectively. In particular, arbitrarily disordered files were repaired using the repair.sh program from BBMap. Pre-processed reads of humans and mice were then aligned to the GRCh38.94 (release 94) or GRCm38 (release 94) reference genome using STAR [35] and subsequently, expression was calculated using RNA-Seq by Expectation Maximization v1.3.1 [36]. Transcripts per kilobase per million (TPM) values were used.

### Identification and analysis of differentially expressed genes (DEGs) between exhausted and non-exhausted CD8+ T cells

The TPM values in each sample were normalized using the up-quartile normalization method. The R package limma was used to remove batch effects. Principal component analysis (PCA) was performed before and after removing the batch effect via FactoMineR and factoextra packages in R. The RP function in the RankProd R package was used to analyze DEGs [37]. The topGene function in the package was used to identify DEGs that were required to pass a False discovery rate (FDR) threshold of 0.05, and a linear fold change threshold of 1.5. Gene ontology (GO) analysis based on the biological process and construction of protein-protein association networks was performed using the Search Tool for the Retrieval of Interacting Genes/Proteins (STRING, https://string-db.org/) database [38]. A process or pathway was considered to be significantly enriched if the FDR value was < 0.05. The protein-protein interaction files obtained from STRING were then used to identify hub genes in the network. The hub genes with the top interaction degrees were extracted and analyzed using Cytoscape software version 3.7.2 [39] and its cytoHubba [40] plugin.

### Co-expression based on weighted correlation network analysis (WGCNA)

WGCNA [41] is an R package that is used to study clusters (modules) of highly correlated genes, the relationship between clinical traits and gene expression profiles, as well as calculating the correlation values within each module or module-trait relationships. The main WGCNA workflow was as follows: (i) The gene expression matrix and clinical traits were prepared and normalized TPM values were used. (ii) The soft thresholding power was determined by calling the network topology analysis pickSoftThreshold function. The set of candidate soft-thresholding powers ranged from 1–30. Then, the scale independence and mean connectivity for each power were calculated. A suitable power was selected if the degree of independence was more than 0.9 (human 11, mouse: 12). (iii) A co-expression network (unsigned network) was constructed and modules were identified. Modules are defined as clusters of highly correlated genes. Unsupervised clustering is used to identify modules, while here the default method is hierarchical clustering by using the standard R function hclust. All genes were assigned to dozens of modules via hierarchical clustering and dynamic tree cutting. These modules were represented using unique color labels. (iv) Modules were related to external information, such as clinical traits. First, the Module eigengene (ME) values were recalculated. An eigengene was the first principal component of a module expression matrix and thus was considered representative information for all genes in each module. Second, these eigengenes were correlated with clinical traits to obtain significant associations using the Pearson coefficient. Finally, the module-trait relationships were visualized using the labeledHeatmap function. (v) The weight was added to the existing module eigengenes and the relationships among eigengenes and the exhaustion trait were plotted using the plotEigengeneNetworks function, including a heatmap and dendrogram. (vi) The module membership (MM) was examined and the key drivers in interesting modules were identified. First, a matrix of Pearson correlation coefficients was obtained among genes and modules. Second, a similar matrix among genes and exhaustion traits was obtained. Third, the two matrices were combined and the interesting modules were analyzed and visualized using the verboseScatterplot function.

### Gene set enrichment analysis (GSEA)

GSEA was performed using the GSEA software version 4.0.2 [42]. Eighty gene sets associated with T cell exhaustion traits were collected from the molecular signatures database v7 (MSigDB 7.0) and used for GSEA. Kyoto encyclopedia of genes and genomes (KEGG) pathway enrichment analysis was performed using GSEA. The gene set used was c2.cp.kegg.v7.0.symbols.gmt. The normalized enrichment score and FDR values were calculated using permutation testing.

### Statistical analysis

Statistical analyses were performed using R software, version 3.5.2. Statistical analysis was performed using an unpaired two-tailed Student’s *t*-test (*P < 0.05, ** *P* < 0.01, *** *P* < 0.001).

## Results

### Human and mouse samples were characterized using RNA-seq datasets

**Fig 1:**
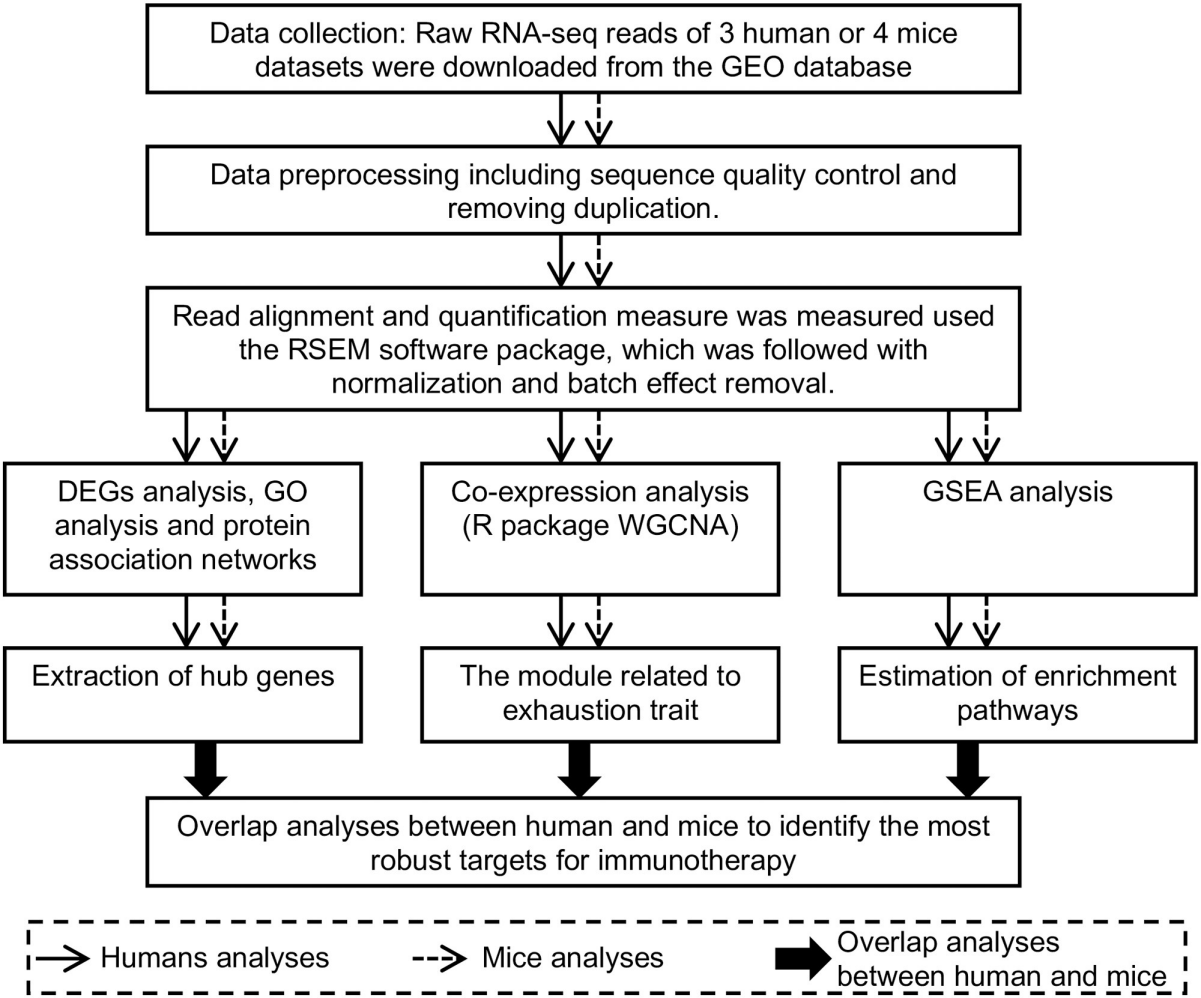
A schematic diagram of the workflow. GEO, Gene expression omnibus; RSEM, RNA-Seq by Expectation–Maximization; DEGs, Differentially expressed genes; GO, Gene ontology; WGCNA, Weighted correlation network analysis; GSEA, Gene set enrichment analysis.

The analysis procedure used in this study is shown in Fig 1. Raw RNA-seq reads were collected from seven studies, including three *Homo sapiens* and four *Mus musculus* studies. CD8+ T cell samples with exhausted or non-exhausted traits were selected according to rigid criteria (see the Materials and Methods). A total of 88 human and 35 mouse samples showing either exhausted or non-exhausted traits were used to comprehensively uncover the molecular mechanisms of CD8+ T cell dysfunction and identify highly efficient targets for the treatment of cancer and chronic infections (Table 1). Detailed information of the 123 samples is shown in S1 Table.

**Table 1:** *Homo sapiens* and *Mus musculus* sample category information.

Species	Status	Sample size	Reference
*Homo sapiens*	Exhausted	11	Thommen et al. (2018)
	Non-exhausted	26	
	Exhausted	6	Kim et al. (2018) [27]
	Non-exhausted	12	
	Exhausted	16	Long et al. (2016)
	Non-exhausted	17	
*Mus musculus*	Exhausted	6	Mognol et al. (2017)
	Non-exhausted	12	
	Exhausted	2	Man et al. (2017)
	Non-exhausted	4	
	Exhausted	3	Utzschneider et al. (2016)
	Non-exhausted	3	
	Exhausted	5	Pauken et al. (2016)
	Non-exhausted	0	

In this study, virus- and tumor-specific CD8+ T cells became exhausted within infections, cancer, or other disease microenvironments, such as LCMV infection, non-small-cell lung cancer, hepatocellular carcinoma, and T1D. Raw reads were then aligned to human or mouse reference genomes separately and TPM values were calculated (see the Materials and Methods section for more information). To compare gene expression profiles of exhausted and non-exhausted CD8+ T cells that were collected from different studies and diseases, batch effects were removed after TPM value normalization. PCA analysis was further performed to compare overall profiles before and after batch effect removal and normalization (S1 Fig). Original datasets with different resources were classified into different clusters for humans and mice; however, there were no obvious clusters after batch effect removal and normalization. Samples with different exhausted and non-exhausted traits were divided into different clusters after batch effect removal and normalization, indicating that the gene expression profiles showed differences due to these traits.

### DEGs of exhausted and non-exhausted CD8+ T cells

**Fig 2:**
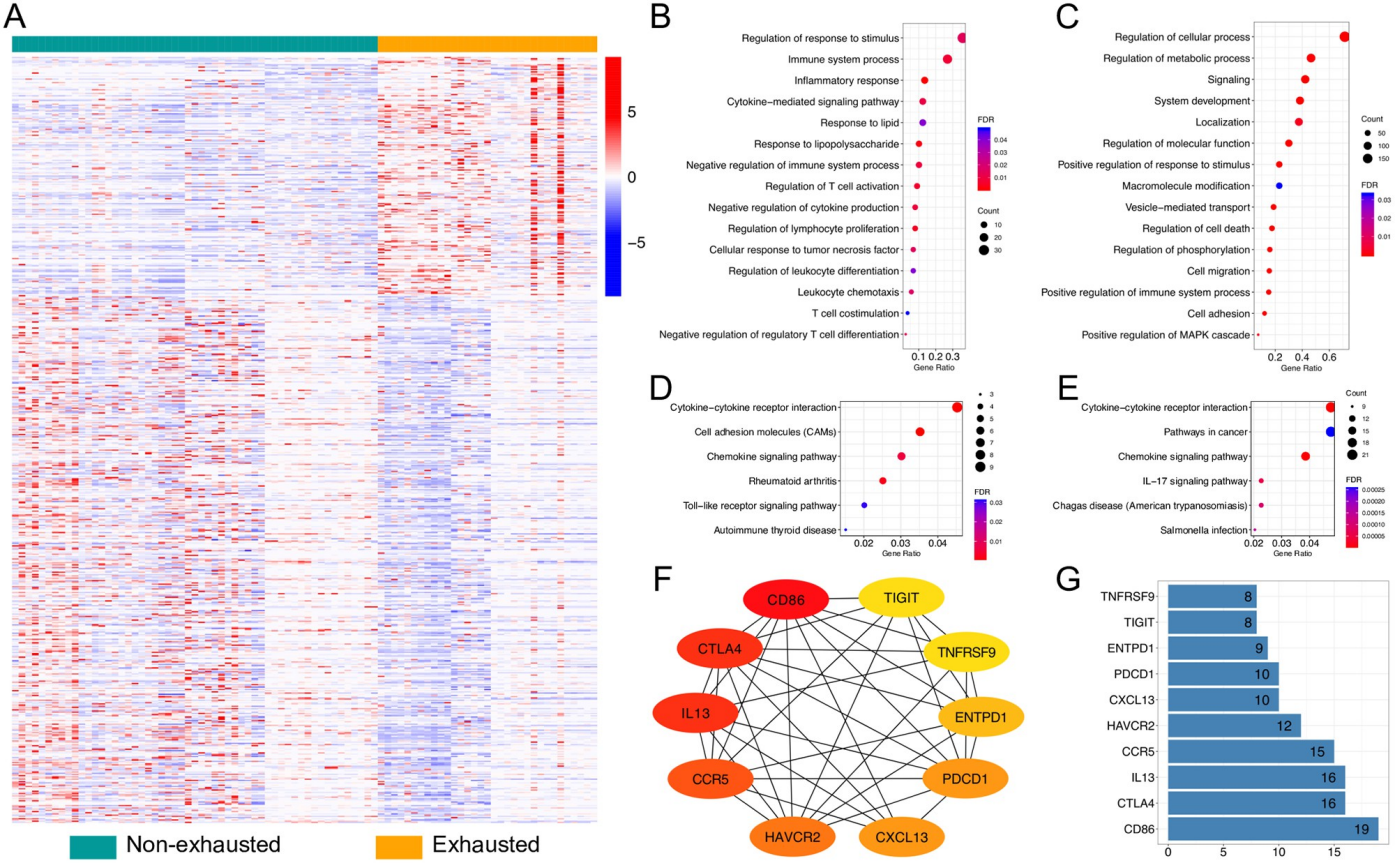
Human differentially expressed gene (DEG) analysis. (A) Heatmap constructed using 199 upregulated and 441 downregulated genes in exhausted T-cells. Significant changes are determined as fold change > 1.5 and False discovery rate (FDR) < 0.05. (B–C) Gene ontology (GO) and (D–E) Kyoto encyclopedia of genes and genomes (KEGG) pathway enrichment analysis for human DEGs. Selected key biological processes in (B) upregulated and (C) downregulated genes. GO analysis is performed based on biological process. (D) All six enriched KEGG pathways in upregulated genes. (E) The top six enriched KEGG pathways in downregulated genes. GO terms and KEGG pathway remain when the FDR is < 0.05. (F–G) Functional protein association networks of upregulated genes. (F) Cytoscape software and Cytohubba plugin have been used to identify hub genes with top 10 interaction degrees via analyzing interactions files obtained from the Search Tool for the Retrieval of Interacting Genes/Proteins (STRING) database. Red to yellow represents interaction degrees from top to bottom. (G) Interaction degrees of the hub genes. X-axis represents the number of adjacent genes.

Having characterized the exhausted and non-exhausted CD8+ T cells, DEGs associated with exhausted CD8+ T cells were identified using normalized TPM values. Genes showing significantly different expression were defined as fold change > 1.5, and FDR < 0.05. A total of 199 upregulated and 441 downregulated genes were identified in exhausted CD8+ T cells in humans (S2 Table). Distinct expression profiles of these DEGs in both exhausted and non-exhausted CD8+ T cells were displayed and showed distinct patterns between exhausted and non-exhausted samples (Fig 2A). Compared to humans, 546 upregulated and 575 downregulated genes were identified in exhausted CD8+ T cells of mice (S2 Table). Subsequently, a heatmap of the 1,121 DEGs was constructed and showed obvious gene signatures in exhausted datasets, which were markedly distinct from non-exhausted datasets (S2A Fig).

Next, the DEGs in exhausted CD8+ T cells were used to identify enriched GO terms (biological processes) and KEGG pathways (Fig 2B–2E, S2B and S2C Fig, and S2 Table). Upregulated genes were involved in several GO biological processes and KEGG pathways, such as inflammatory response, regulation of cytokine production, immune system process, and lymphocyte proliferation. Specifically, several cytokines, immune-related molecules, and chemokines were upregulated in exhausted T cells and involved in the processes or pathways, such as CCL3 (also known as MIP-1a), CCL4L1, TNF (tumor necrosis factor) and receptor family genes (TNFSF4 and TNFRSF9), CXCL13 (also known as C-X-C motif chemokine ligand-13), IL13. Of particular note, the inhibitory receptors PD1, CTLA4, HAVCR2 (also known as T-cell immunoglobulin, and mucin-domain containing-3 [TIM3]), and TIGIT were also among the most upregulated genes in exhausted T cells. Downregulated genes were enriched in signaling, cell migration, cell adhesion, and regulation of phosphorylation. We found that CXCL chemokine family, such as CXCL1, CXCL2, CXCL3, CXCL8, and CXCL16, were enriched, which might imply their roles in T cell exhaustion. In particular, exhausted CD8+ T cells display suppressed IFN-γ production [2, 43], whereas here, its receptor, IFN-γ receptor 2 (IFNGR2), was expressed at a low level among exhausted samples, which might affect the IFN-γ signaling activation. In contract, it has been reported that some exhausted subsets expressed a decent amount of IFNg [44], thus we hypothesized that this might be due to the severity of T cell exhaustion as the impairment of IFN-γ production occurs in the later stage of exhaustion [45, 46]. Functional enriched KEGG pathway analysis indicates that human DEGs are enriched in several pathways, as shown in Fig 2D and 2E. Among the total enriched pathways of upregulated genes, cytokine-cytokine receptor interaction, rheumatoid arthritis, and chemokine signaling pathways were also enriched in downregulated genes. Cell adhesion molecules, autoimmune thyroid disease, and Toll-like receptor signaling pathways were unique to upregulated genes. Intriguingly, the IL-17 signaling pathway is predominantly enriched in downregulated genes. In detail, we found that the CXCL chemokine family (CXCL1, CXCL2, CXCL3, CXCL8), FOS, CCL2, IL1B, MMP9, S100A9, and TRAF4 were enriched in this pathway, but most of them were associated with chemokines and cell proliferation rather than the IL-17 production.

We further analyzed protein association network of upregulated genes by using the STRING website [38]. To identify the key drivers of the networks, the Cytoscape Cytohubba plugin [39, 40] was used to identify hub genes with the top 10 or 75 interaction degrees by analyzing interactions files obtained from the STRING database (Fig 2F and 2G, S2D and S2E Fig). Among the hub genes, three of the key driver genes, programmed cell death protein 1 (*PDCD1*), *HAVCR2*, and *TNFRSF9* (its alternative name is CD137), were simultaneously identified in both humans and mice. In addition, two main networks were observed in mice (S2D Fig). One network was related to the immune system process, containing genes, such as PD1, TIGIT, HAVCR2, and TNF superfamily member 4 (TNFSF4). Another main network, which contained interactions supported by experiments (shown in magenta), was associated with the mitotic cell cycle process and cell population proliferation, such as maternal embryonic leucine zipper kinase (MELK) and mitotic checkpoint serine/threonine-protein kinase BUB1 beta (BUB1B), encoded by *MELK* and *BUB1B*, respectively. Of note, some genes that may play important roles in exhaustion were not connected to the identified networks because only the hub genes with top 10 or 75 interaction degrees were shown, such as adhesion G protein-coupled receptor G1 (ADGRG1), thymocyte selection associated high mobility group box (TOX/TOX2), and CD200R/CD200R1.

### Identification of key genes in exhausted CD8+ T cells based on WGCNA

**Fig 3:**
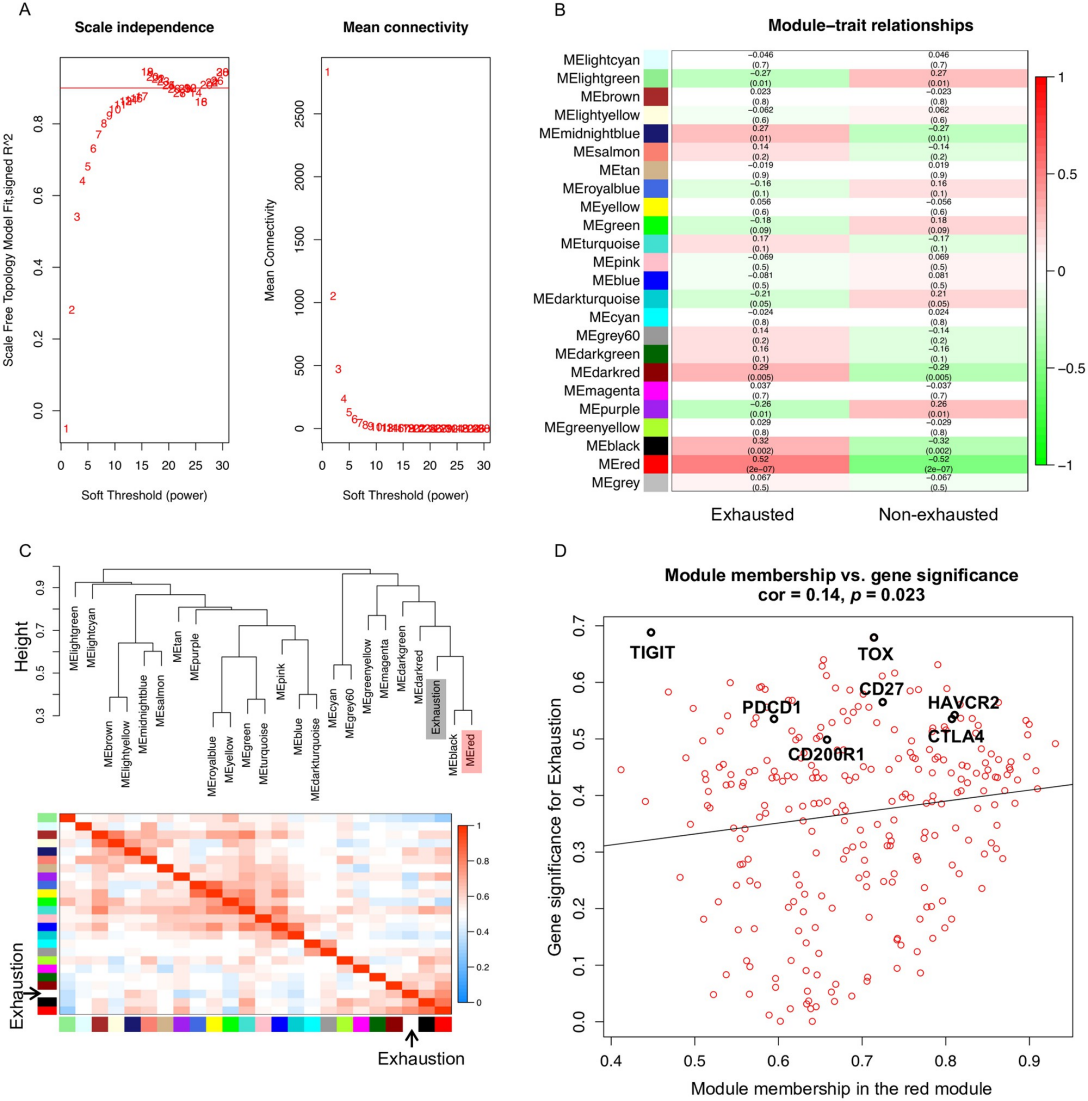
Co-expression analysis in human exhausted T-cells. (A) Analysis of a set of soft thresholding powers. The left panel is the scale free fit index as a function of soft thresholding power. The right panel is mean connectivity as a function of the soft thresholding power. (B) Heatmap of module and trait correlation. Module is marked by unique color labels. ME: module eigengene. (C) Eigengene dendrogram and heatmap between modules and the exhaustion trait. The top figure represents the hierarchical clustering dendrogram of MEs and exhaustion trait. The bottom figure shows the eigengene network heatmap plot of the adjacencies including exhaustion trait. Each row and column corresponds to one ME or exhaustion trait. Red color represents positive correlation, while blue represents negative correlation. (D) Scatterplot of gene significance for exhaustion trait (y-axis) vs. membership in a selected module (x-axis).

WGCNA is an effective method to detect co-expressed modules and hub genes in many aspects [47]. To investigate the relationship between the modules and clinical traits and identify potential prognostic markers of exhausted T cells, co-expressed modules were constructed with WGCNA using the gene expression profiles of exhausted and non-exhausted CD8+ T cells in humans (Fig 3) and mice (S3A–S3D Fig). A total of 11,499 genes from 88 human samples were used for WGCNA. A suitable power of 11 was selected for the co-expression analysis (Fig 3A). Genes with common biological functions and associations were assigned into 24 co-expressed modules (clusters), including the gray module, which contained the genes that were not classified into any of the other 23 modules. Subsequently, the correlation values between the modules and clinical traits (exhausted or non-exhausted) were calculated based on the correlations between module eigengenes (MEs) and clinical traits, demonstrating that the red module was most positively correlated with exhaustion and that the correlation value was 0.52 (Fig 3B). To detect groups of correlated eigengenes and exhaustion, the existing 23 modules (except the gray module) were further analyzed using eigengene dendrogram and eigengene adjacency heatmap (Fig 3C). The dendrogram also suggested that the red module was highly related to T cell exhaustion. In addition, the eigengene dendrogram showed a certain correlation between the black module and exhaustion, but its correlation value (0.32) was less than the red module (Fig 3B). Taken together, the genes in the red modules, including 263 genes, might play a critical role in T cell exhaustion (S3 Table) and need further studies. Next, to measure the importance of each gene within a specific module and their contributions to the phenotype, the module membership (MM) vs. gene significance (GS) of the interesting red module was calculated (Fig 3D). The MM correlates the MEs with gene expression values and implies how close a gene is to a given module, while the GS represents the correlation between individual genes and trait (T cell exhaustion) [41]. Generally, if a gene exhibit high levels of MM and GS values, it may represent an important membership for the module and is significantly related to the trait. Notably, the well-studied inhibitory receptors, PD1, CTLA4, HAVCR2, TIGIT, and CD27 (a member of the tumor necrosis factor receptor superfamily), showed a high level of MM vs. GS, indicating that they were highly correlated with T cell exhaustion and key components of the underlying biological function.

**Fig 4:**
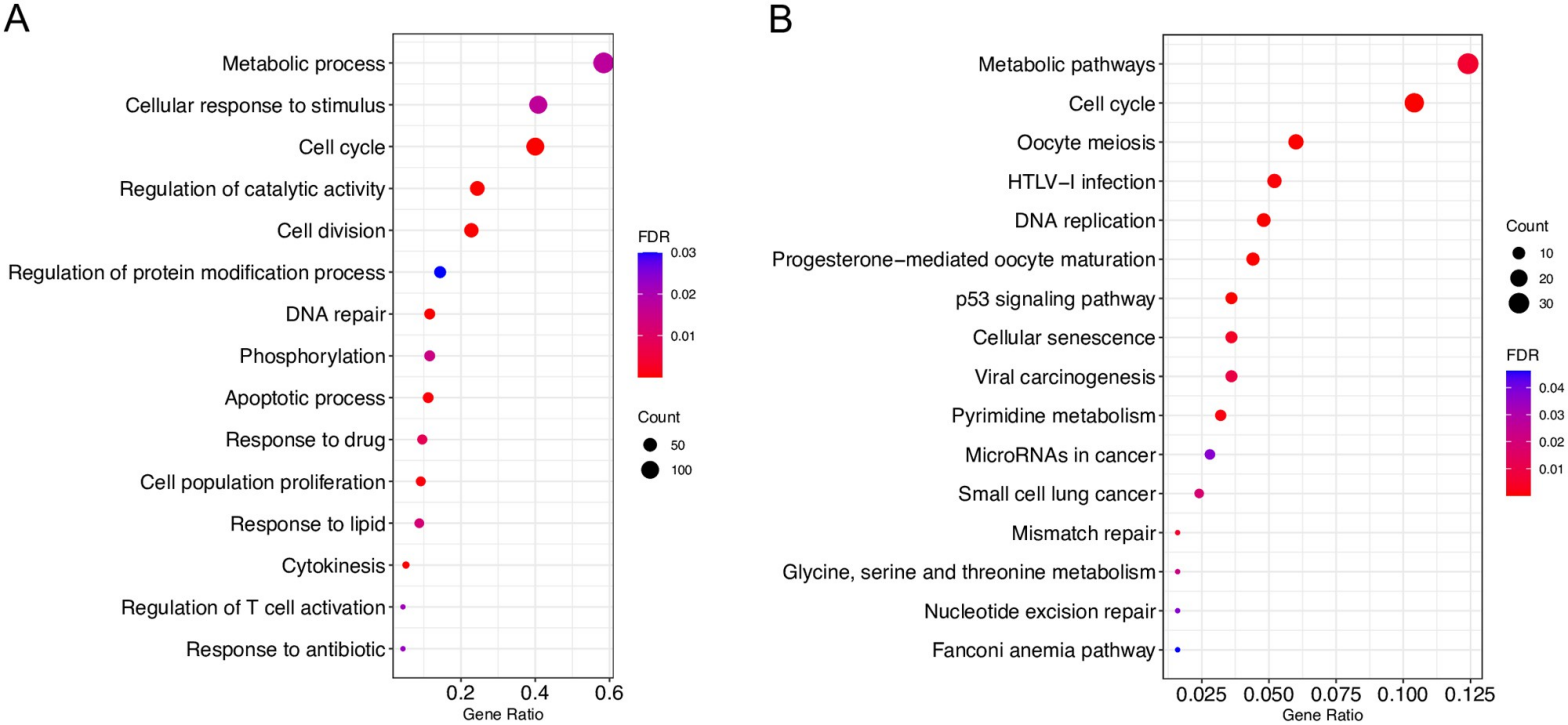
GO and KEGG pathway enrichment analysis for human red module genes. (A) Selected key biological processes. (B) All enriched KEGG pathways. GO terms and KEGG pathway remain when the FDR is < 0.05.

Genes in the red module were further used for GO (biological process) and KEGG pathway enrichment analyses (Fig 4). Metabolic derangements occur in exhausted T cells [2]. Here, the GO and KEGG pathway analyses demonstrated that these genes were particularly enriched in metabolic pathways (such as pyrimidine metabolism and glycine, serine, and threonine metabolism), cell cycle, DNA replication, phosphorylation, and p53 signaling pathway, which may shed light on T cell exhaustion mechanisms.

### Upregulations of CD200R1 and ADGRG1 in exhausted CD8+ T cells

**Fig 5:**
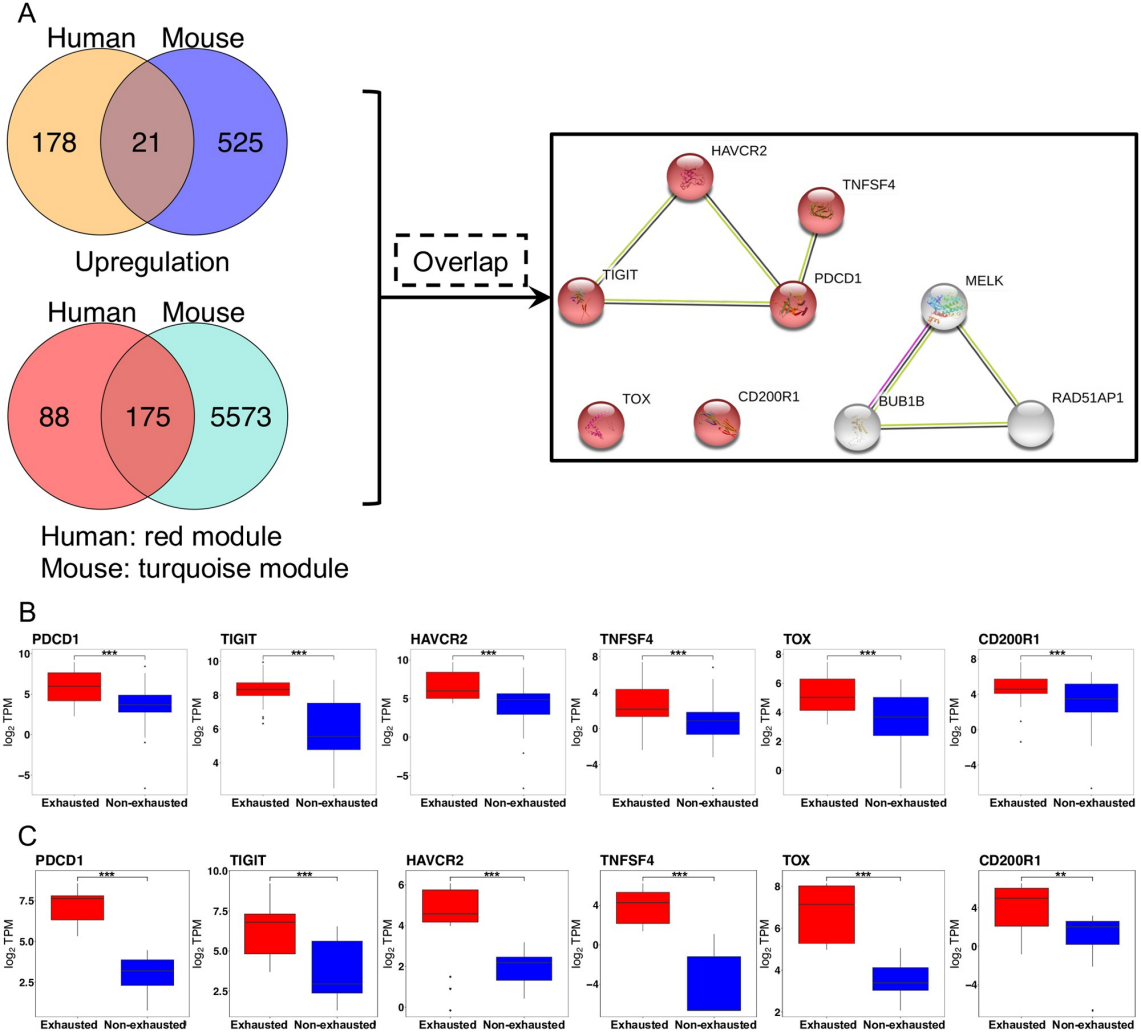
Overlap genes between human and mouse sets. (A) Overlap of upregulated genes in exhausted T-cells and most correlative modules with exhaustion between human and mouse, as well as protein associated network of nine proteins in both the overlap of upregulated genes and selected modules. Red colored nodes: regulation of immune system process (GO:0002682). (B) and (C) show the expression of selected genes at the mRNA level in human and mouse datasets from exhausted (red) and non-exhausted (blue) T cells, respectively. Median values are indicated by lines in the box and whisker plot. Hinge values and whisker 1.5* interquartile range (IQR) values have been calculated using Tukey. Statistical analysis has been performed using an unpaired two-tailed Student’s *t*-test (*P < 0.05, ** *P* < 0.01, *** *P* < 0.001).

Next, to investigate the novel and robust driver genes in T cell exhaustion that caused by chronic infection or other diseases, the above findings from the upregulated genes and co-expression analyses (Fig 5A) were integrated to discover the underlying molecular mechanisms. First, 21 overlapping upregulated genes were identified between humans and mice. Second, 175 overlapping genes were identified from the most exhausted-correlated modules (human, red module; mouse, turquoise module) based on co-expression analysis. Eventually, nine overlapping genes were obtained by combining the upregulated genes and co-expression analyses between humans and mice. The nine overlapping genes were further used to construct protein-protein association networks using the STRING database. Six of the nine proteins [PD1 (encoded by *PDCD1*), TIGIT, HAVCR2, TNFSF4 (also known as CD134 or OX40L), TOX, and CD200R1] were associated with the regulation of immune system processes. In particular, the well-studied PD1, TIGIT, HAVCR2, and TNFSF4 were classified into one of the main networks, while TOX and CD200R1 belonged to neither of the main networks. Of particular note, *TOX* and *CD200R1* showed a high level of MM vs. GS, suggesting that these two genes were most important during T cell exhaustion (Fig 3D). In addition, the expression profiles of the six genes were confirmed at the mRNA level (Fig 5B and 5C). Compared to non-exhausted CD8+ T cells, the expression of *TOX* and *CD200R1* was similar to that of the well-studied inhibitory receptors, which showed distinctly high levels of expression in exhausted CD8+ T cells.

However, many recent studies have reported TOX as a promoting factor for T cell exhaustion [29, 30, 48, 49], while CD200R1 is a favorable prognostic factor [50], suggesting that those samples may show less exhaustion presumably. To further demonstrate that the CD200R1, TOX, and other well-studied genes indeed contribute to T cell exhaustion rather than due to certain diseases and technique factors, we reconducted the WGCNA analysis by removing the T1D dataset (anti-CD3 therapy) (S4 Fig) and confirmed the expressions of corresponding genes in each dataset (S5 Fig). In detail, the WGCNA analysis revealed the highest positive correlation between exhaustion and the brown module. Most importantly, all the six overlapping genes of interest in Fig 5 were also identified in the brown module, suggesting that those genes’ contributions to T cell exhaustion may not be affected by the T1D datasets. Further visualization of the six gene expressions showed that CD200R1 and other genes were considerably upregulated in each dataset, respectively, demonstrating that the gene upregulations were not driven by any specific dataset.

Furthermore, we found that ADGRG1 was also considerably upregulated in both human and mouse exhausted T cells (S6 Fig), but was only co-expressed in the selected mouse turquoise module, and not in the human red module. G protein-coupled receptor 56 (GPR56) is encoded by ADGRG1 [51] and is expressed in all human cytotoxic lymphocytes [52].

Collectively, the transcriptome analysis of human and mouse exhausted CD8+ T cells revealed that the six genes played important roles in CD8+ T cell exhaustion. Of particular note, most of their contributions to exhaustion are well known, but the roles of CD200R1 and ADGRG1 are lesser described in previous studies. Those two targets that we identified may shed light on well understanding of T cell exhaustion by further in vitro tests.

### Predefined exhaustion gene sets were enriched in exhausted CD8+ T cells

**Fig 6:**
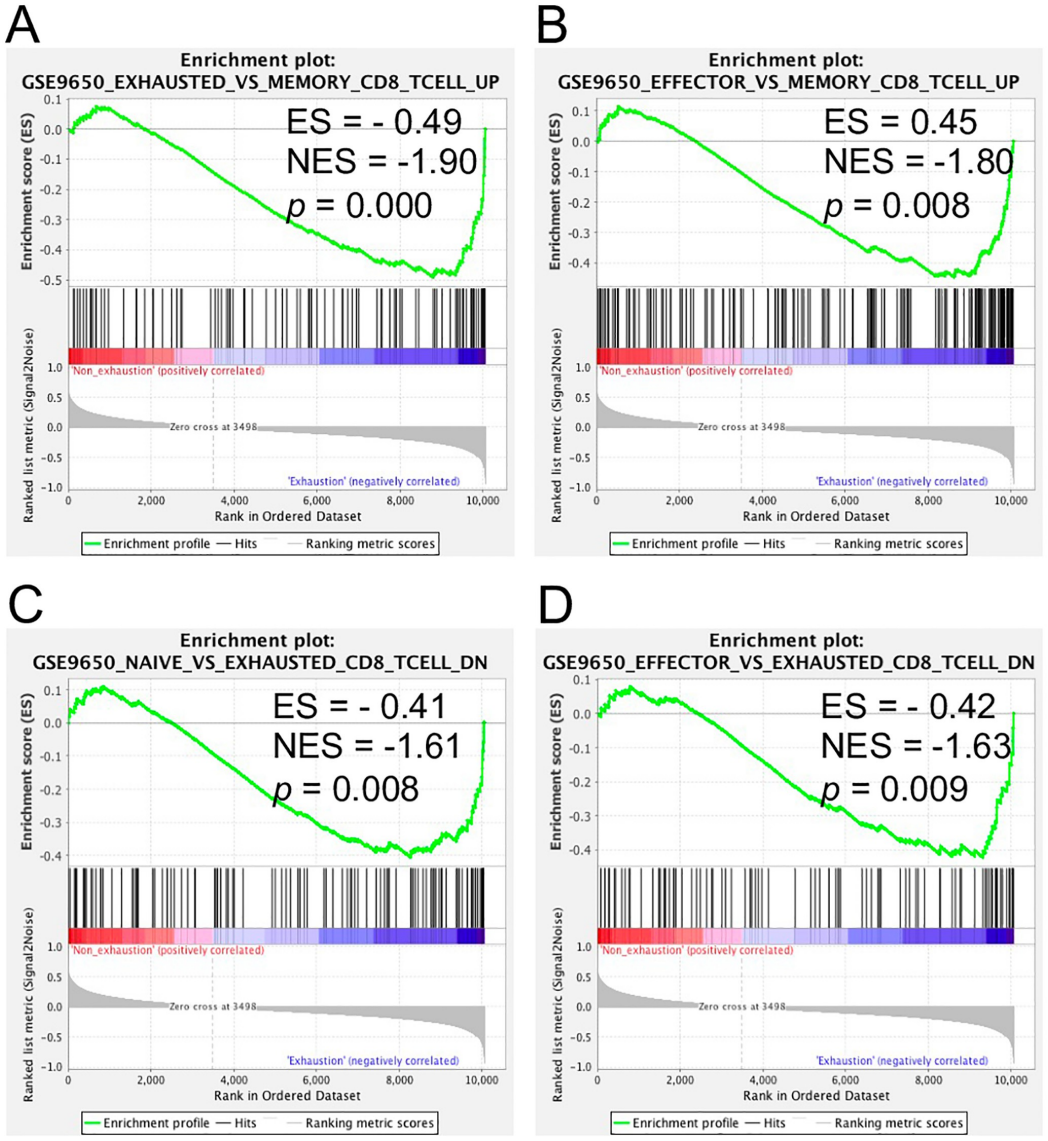
GSEA of exhausted versus non-exhausted CD8+ T-cells. (A–D) The four shared enriched gene sets in human exhausted T-cells with a *P*-value < 0.05. ES, enrichment score; NES, normalized enrichment score.

Additionally, to interpret genome-wide transcriptional profiles of the exhausted T cell samples in this study and further examine the molecular mechanisms of T cell exhaustion, GSEA was performed using the 80 predefined gene sets collected from MSigDB that contained information on immunologic signatures, and gene sets from the biomedical literature. The 80 gene sets were associated with CD8+ T cell exhaustion during chronic infection or cancer. Compared to 17 gene sets in mice, 12 of 80 gene sets were significantly enriched in exhausted CD8+ T cells in humans (*P* < 0.05) (S4 Table). The gene members of the majority of those enriched gene sets were either upregulated or downregulated in comparison to exhausted or PD1 high CD8+ T cells versus effector, naïve, PD1 low, or memory CD8+ T cells. Four enriched gene sets in the exhausted CD8+ T cells were shared between humans and mice (Fig 6 and S3E–S3H Fig), including gene sets GSE9650_effector_VS_exhausted_CD8_TCELL_DN (genes downregulated in comparison to effector CD8 T cells versus exhausted CD8 T cells), GSE9650_naïve_VS_exhausted_CD8_TCELL_DN (genes downregulated in comparison to naive CD8 T cells versus exhausted CD8 T cells), GSE9650_effector_VS_memory_CD8_TCELL_UP (genes upregulated in comparison to effector CD8 T cells versus memory CD8 T cells), GSE9650_exhausted_VS_memory_CD8_TCELL_UP (genes upregulated in comparison to exhausted CD8 T cells versus memory CD8 T cells). These gene sets were obtained from the MSigDB C7 immunologic signatures [21]. Most of the genes in the four gene sets were categorized as transcription factors, cell differentiation markers, oncogenes, cytokines, and growth factors. Additionally, during CD8+ T cell exhaustion caused by either chronic infection or other diseases in this study, some DEGs of human were particularly identified in the four enriched gene sets (S5 Table). These findings may provide insight into understanding the consistent mechanisms of T cell exhaustion caused by multiple factors between human and mice.

## Discussion

Immunotherapies that reactivate exhausted T cells by targeting immune checkpoint inhibitors have emerged as promising treatment options [16, 17, 53, 54]. Understanding the features of and pathways to T cell exhaustion has crucial implications for successfully blocking immune checkpoint inhibitors and immunotherapies. However, T cell exhaustion mechanisms and checkpoint immunotherapy are yet to be fully described. As T cell exhaustion has been separately described in humans and mice during chronic infection, cancer, HIV, or hepatitis C virus infection [10–12, 55], in this study, both human and mouse exhausted CD8+ T cells were comprehensively analyzed. As exhaustion may display potential differences between cancer and chronic infection, the exhausted T cells used in this study were developed during multiple microenvironments, such as non-small-cell lung cancer, hepatocellular carcinoma, T1D, or chronic infections with seven origins. Above all, these data might be able to shed light on the mechanisms of exhaustion.

The independent transcriptome analyses of humans and mice indicated that several genes, especially CD200R1 and TOX, were significantly upregulated in exhausted CD8+ T cells and were key drivers of the modules that contributed to the exhaustion trait based on the WGCNA. Recent studies have also highlighted the important role of the TOX transcriptional regulator in driving T cell exhaustion and diminishing anti-tumor or anti-viral functions [56–58]. In this study, we found that CD200R1 showed important role in exhaustion. Previous study has been reported that it was considered a viable therapeutic target in cancer by blocking CD200R in the CD200R1 pathway [54]. Compared to CD200R1 and TOX, GPR56 might also be a candidate target, even though its robustness is weaker (not detected in the human red module).

The mechanisms underlying T cell exhaustion were uncovered. Hallmarks of exhausted T cells include overexpression of inhibitory receptors, loss of proliferative potential and cytokine production, markedly different transcriptional profiles, metabolic derangement, and the use of key transcription factors [2, 12, 21, 55, 59, 60]. These findings might explain and demonstrate the formation of these key characteristics. First, the inhibitory receptors, PD1, CTLA4, TIGIT, HAVCR2, and TNFSF4 were upregulated. Second, several biological pathways were modulated via either significantly upregulated or downregulated genes, which potentially indicated impaired T cell capacity to proliferate and secrete cytokines. Specifically, the GO and KEGG pathway enrichment analyses suggested that DEGs were enriched in cytokine-cytokine receptor interaction, immune system process, cell cycle, phosphorylation, and the IL-17 signaling pathway. Of note, only one pathway, cytokine-cytokine receptor interaction, was enriched in mouse upregulated genes. In addition, the enriched IL-17 signaling pathway in human downregulated genes might provide a novel way to understand T cell exhaustion. Third, co-expressed genes based on WGCNA demonstrated altered metabolism in exhausted T cells. Only protein-protein interaction network and KEGG enrichment analyses of selected human red modules were performed, as there were too many genes in the mouse turquoise module (5748 genes). Thus, genes in the human red module were enriched in metabolic pathways, such as pyrimidine metabolism and glycine, serine, and threonine metabolism. In fact, cell cycle, DNA replication, nucleotide excision repair, and p53 signaling pathways were enriched in genes in the red module. Moreover, genes associated with cell cycle regulation and mitotic checkpoint, including cyclin dependent kinase 1, cyclin B1, budding uninhibited by benzimidazoles 1 (*BUB1*), cyclin A2, mitotic arrest deficient 2 like 1, *BUB1B*, cell division cycle 20, and cyclin B2 were the hub genes of the protein interaction network. Among the eight hub genes, only *BUB1* and *BUB1B* were significantly upregulated.

Additionally, according to the GSEA, a minority of the considerably upregulated or downregulated genes identified in this study were observed in the gene members of the four shared gene sets (S5 Table). Specifically, to our surprise, upregulated *ADGRG1* was identified from two shared gene sets, effector_VS_exhausted_CD8_TCELL_DN and exhausted_VS_memory_CD8_TCELL_UP, which supported our previous hypothesis that ADGRG1 was a potential target for treatment. The downregulated genes, annexin A1 (*ANXA1*) and interleukin 12 receptor, beta 2 subunit (*IL12RB2*) were observed in the gene set effector_VS_memory_CD8_TCELL_UP. ANXA1 and IL12RB2 play important roles in Th1 cell differentiation and are thought to contribute to inflammatory responses and host defense [61, 62]. The legumain gene is downregulated in exhausted_VS_memory_CD8_TCELL_UP and may be involved in endogenous proteins for major histocompatibility class II presentation in the lysosomal/endosomal system [63–65].

Taken together, by taking advantage of the transcriptome analyses between humans and mice, a novel way to understand the mechanisms of T cell exhaustion was revealed. For example, we found the important roles of CD200R1 and ADGRG1 in exhaustion. The full gene lists, such as DEGs, may be served as a reference to the immunology community. Although T cell exhaustion has been identified in humans and mice, it may show differences to some degree, such as altered transcriptional profiles and transcriptional factors. Thus, to fully understand molecular mechanisms of T cell exhaustion and immunotherapy using mice as models, further comprehensive transcriptome analyses of multiple datasets that include humans and mice will be required in the future.

## Supporting information

S1 Fig Principal component analysis (PCA) of *Homo sapiens* (A–C) and *Mus musculus* (D–F). (A, D) and (B, E) display clustering based on the dataset source before and after batch effect removal and normalization, respectively. (C, F) shows the clustering of non-exhausted and exhausted traits after batch effect removal.
(TIF)

S2 Fig Mouse gene expression profile of differentially expressed genes (DEGs). (A) Heatmap constructed using 546 upregulated and 575 downregulated genes in exhausted T-cells. (B–C) Gene ontology (GO) enrichment analysis for mouse DEGs. Selected key biological processes in (B) upregulated and (C) downregulated genes. (D–E) Functional protein association networks of upregulated genes. Top 10 and 75 interaction degrees of hub genes are displayed. Red to yellow represents interaction degrees from top to bottom.
(TIF)

S3 Fig Co-expression and Gene set enrichment analysis (GSEA) of mouse. (A–D) Co-expression analysis of mouse exhausted T-cells. (A) Analysis of a set of soft thresholding powers. (B) Heatmap of module and trait correlation. (C) Eigengene dendrogram and heatmap between modules and the exhaustion trait. (D) Scatterplot of gene significance for exhaustion trait (y-axis) vs. membership in a selected module (x-axis). (E–H) Gene set enrichment analysis (GSEA) of exhausted versus non-exhausted CD8+ T-cells. The four shared enriched gene sets in mouse exhausted T-cells with a *P*-value < 0.05. ES, enrichment score; NES, normalized enrichment score.
(TIF)

S4 Fig WGCNA analysis by removing the T1D dataset in human. PCA analysis of the two datasets was performed before (A) and after removing batch effects. (C) Heatmap of modules and trait correction. (D) Eigengene dendrogram and heatmap between modules and the exhaustion trait. (E) Scatterplot of gene significance for exhaustion trait (y-axis) vs. membership in a selected module (x-axis).
(TIF)

S5 Fig Expression level of selected genes in each human dataset, respectively. Median values are indicated by lines in the box and whisker plot. Hinge values and whisker 1.5* interquartile range (IQR) values have been calculated. *P < 0.05, ** *P* < 0.01, *** *P* < 0.001.
(TIF)

S6 Fig Expression level of ADGRG1. (A) and (B) show the expression of ADGRG1 at the mRNA level in human and mouse datasets, respectively. *P < 0.05, ** *P* < 0.01, *** *P* < 0.001.
(TIF)

S1 Table The RNA-seq datasets for *Homo sapiens* and *Mus musculus*. (DOCX)

S2 Table DEGs and their matched genes for each enriched process and pathway in Fig 2 and S2 Fig. (DOCX)

S3 Table Gene list of the red module in Fig 3. (DOCX)

S4 Table Enriched gene sets of *Homo sapiens* and *Mus musculus*. (DOCX)

S5 Table Overlap genes between the four enriched gene sets in Fig 6 and human DEGs. (DOCX)

## Abbreviations

DEG: Differentially expressed gene
FDR: False discovery rate
GEO: Gene expression omnibus
GO: Gene ontology
GS: Gene significance
GSEA: Gene set enrichment analysis
KEGG: Kyoto encyclopedia of genes and genomes
ME: Module eigengene
PCA: Principal component analysis
TPM: Transcripts per kilobase per million
WGCNA: Weighted correlation network analysis
