# Supplementary figures and images for: Transcriptome analysis of Homo sapiens and Mus musculus reveals mechanisms of CD8+ T cell exhaustion caused by different factors
Source: PLoS One. 2022 Sep 9;17(9):e0274494. doi: 10.1371/journal.pone.0274494 (PMC9462770; doi:10.1371/journal.pone.0274494)

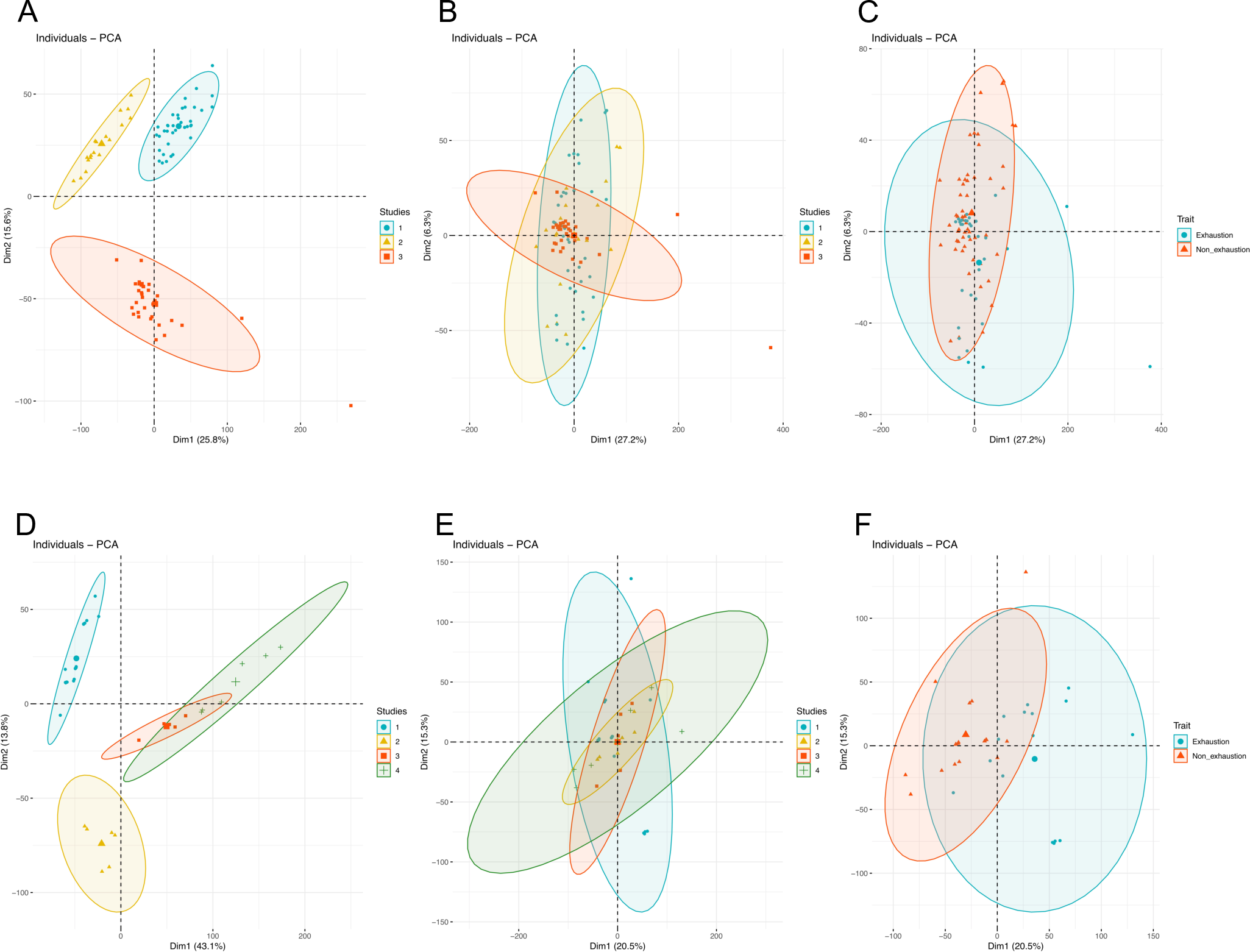

Supplement: S1 Fig — Principal component analysis (PCA) of Homo sapiens (A–C) and Mus musculus (D–F). (A, D) and (B, E) display clustering based on the dataset source before and after batch effect removal and normalization, respectively. (C, F) shows the clustering of non-exhausted and exhausted traits after batch effect removal. (TIF) [file pone.0274494.s001.tif]

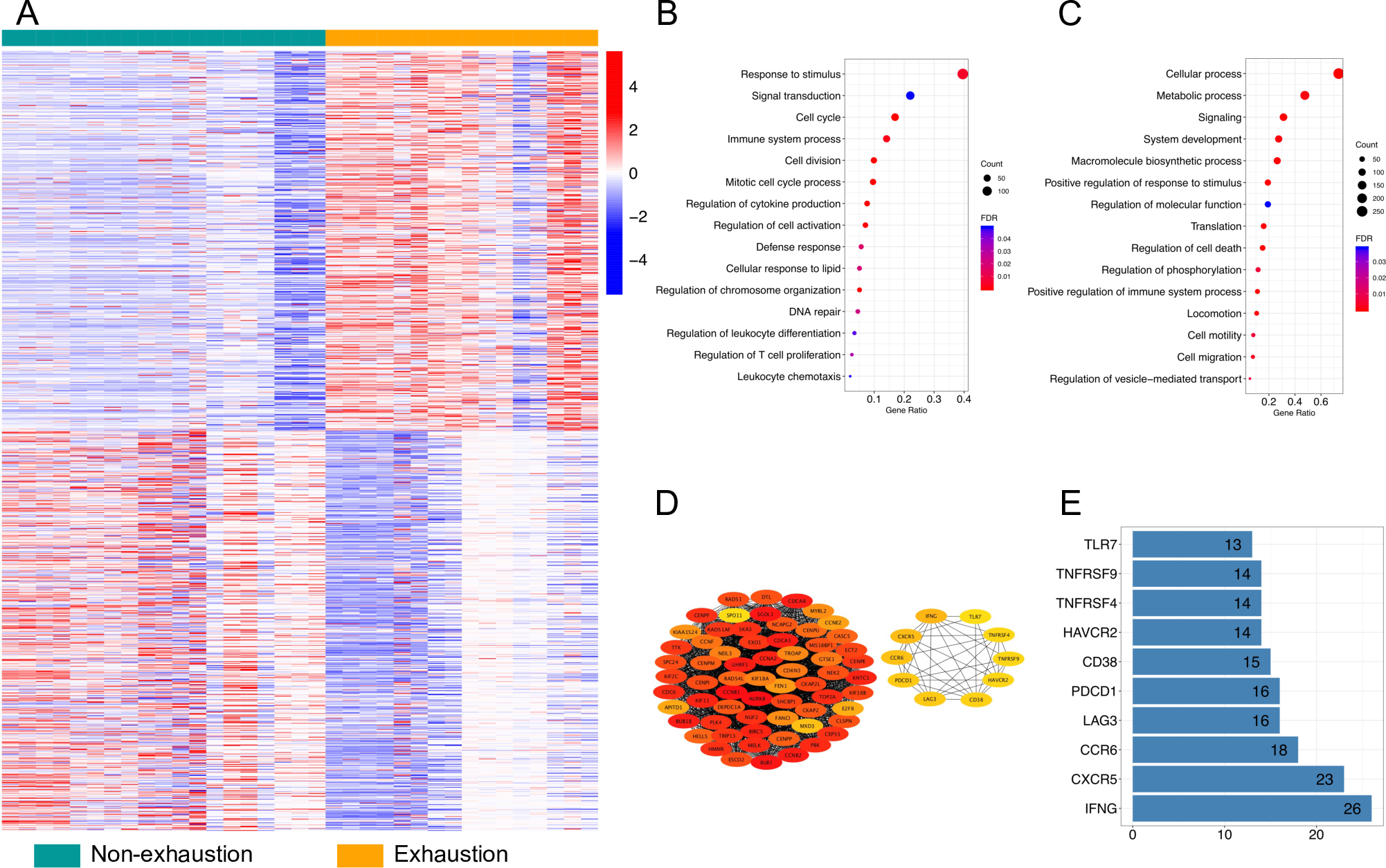

Supplement: S2 Fig — (A) Heatmap constructed using 546 upregulated and 575 downregulated genes in exhausted T-cells. (B–C) Gene ontology (GO) enrichment analysis for mouse DEGs. Selected key biological processes in (B) upregulated and (C) downregulated genes. (D–E) Functional protein association networks of upregulated genes. Top 10 and 75 interaction degrees of hub genes are displayed. Red to yellow represents interaction degrees from top to bottom. (TIF) [file pone.0274494.s002.tif]

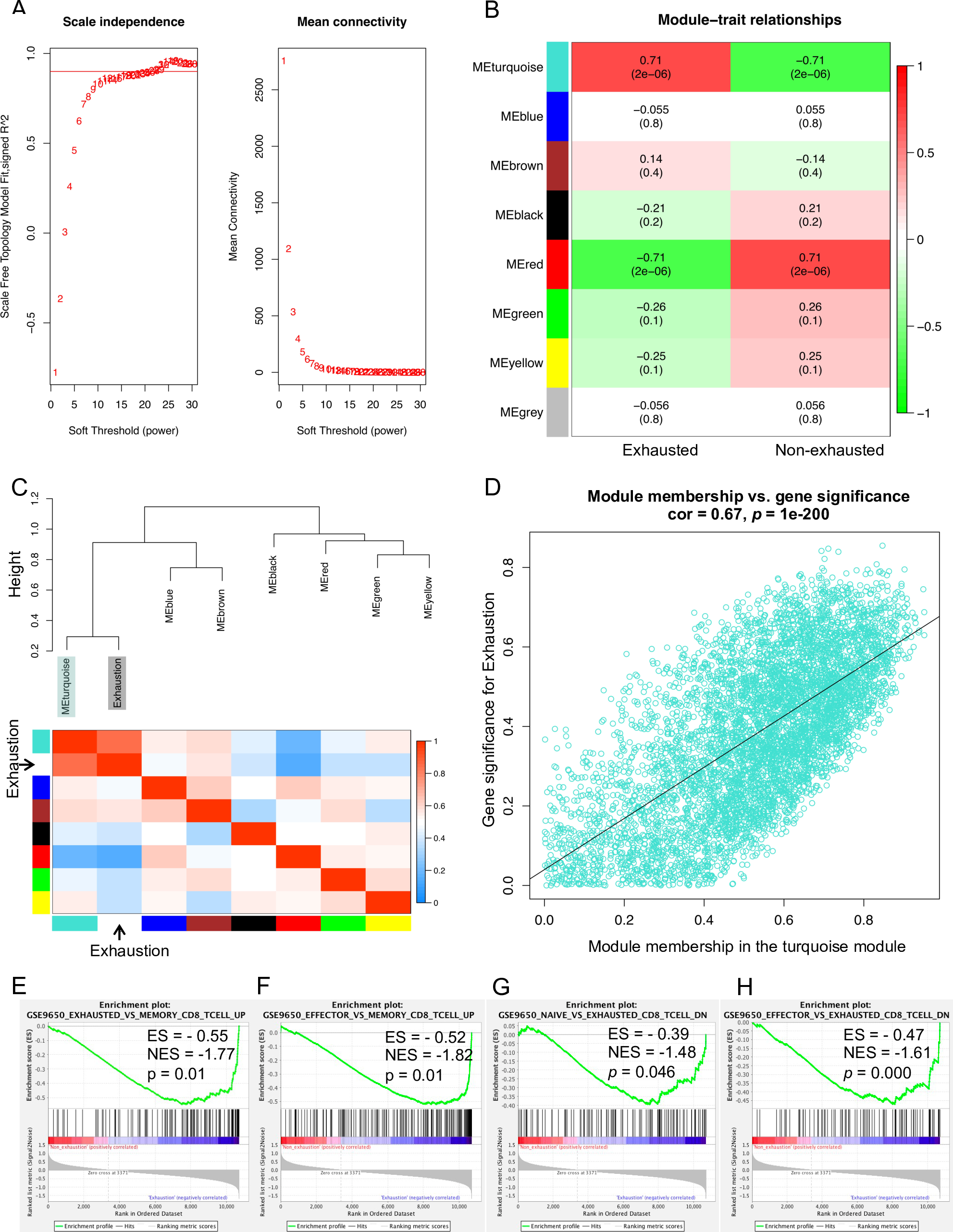

Supplement: S3 Fig — (A–D) Co-expression analysis of mouse exhausted T-cells. (A) Analysis of a set of soft thresholding powers. (B) Heatmap of module and trait correlation. (C) Eigengene dendrogram and heatmap between modules and the exhaustion trait. (D) Scatterplot of gene significance for exhaustion trait (y-axis) vs. membership in a selected module (x-axis). (E–H) Gene set enrichment analysis (GSEA) of exhausted versus non-exhausted CD8+ T-cells. The four shared enriched gene sets in mouse exhausted T-cells with a P-value < 0.05. ES, enrichment score; NES, normalized enrichment score. (TIF) [file pone.0274494.s003.tif]

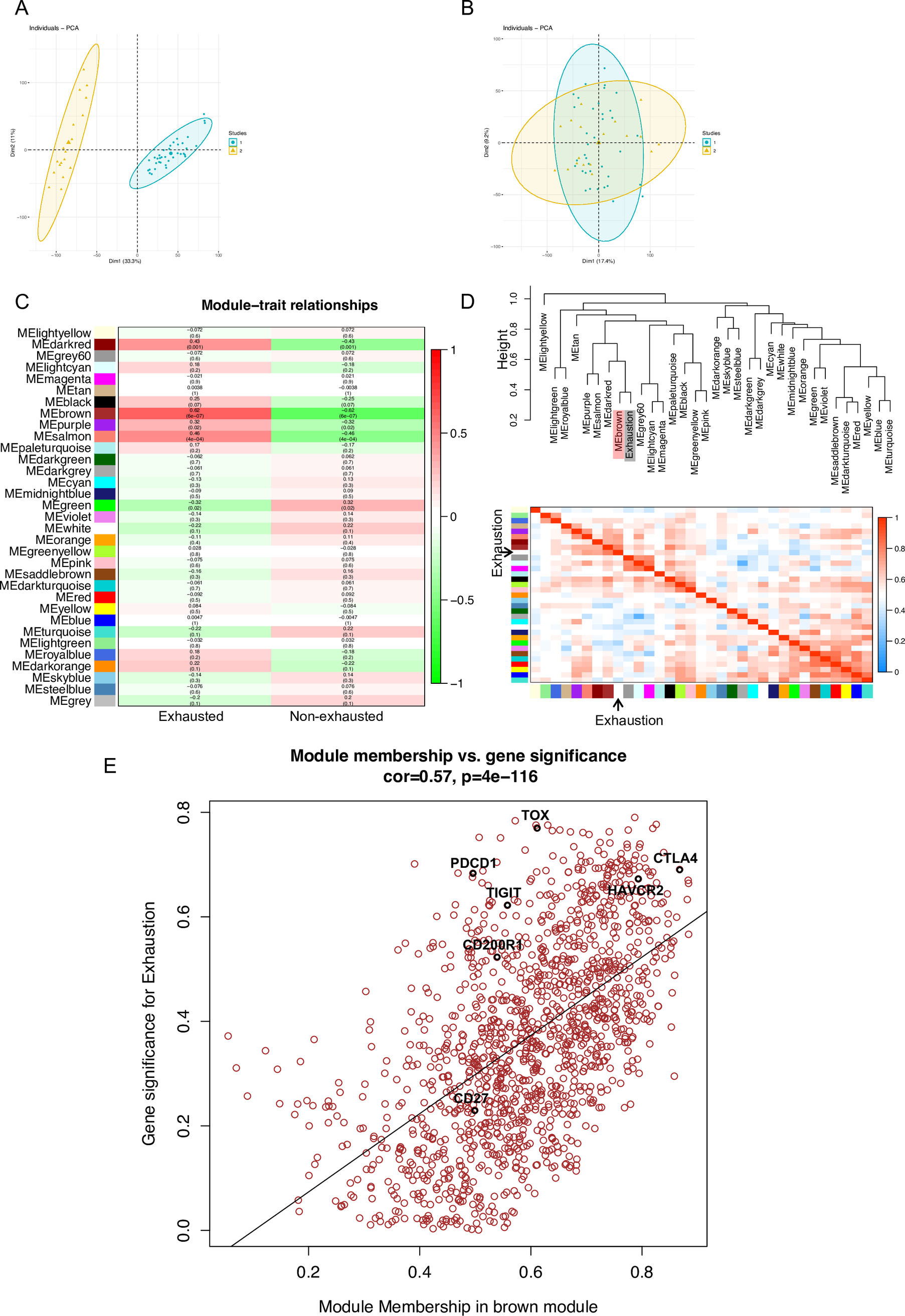

Supplement: S4 Fig — PCA analysis of the two datasets was performed before (A) and after removing batch effects. (C) Heatmap of modules and trait correction. (D) Eigengene dendrogram and heatmap between modules and the exhaustion trait. (E) Scatterplot of gene significance for exhaustion trait (y-axis) vs. membership in a selected module (x-axis). (TIF) [file pone.0274494.s004.tif]

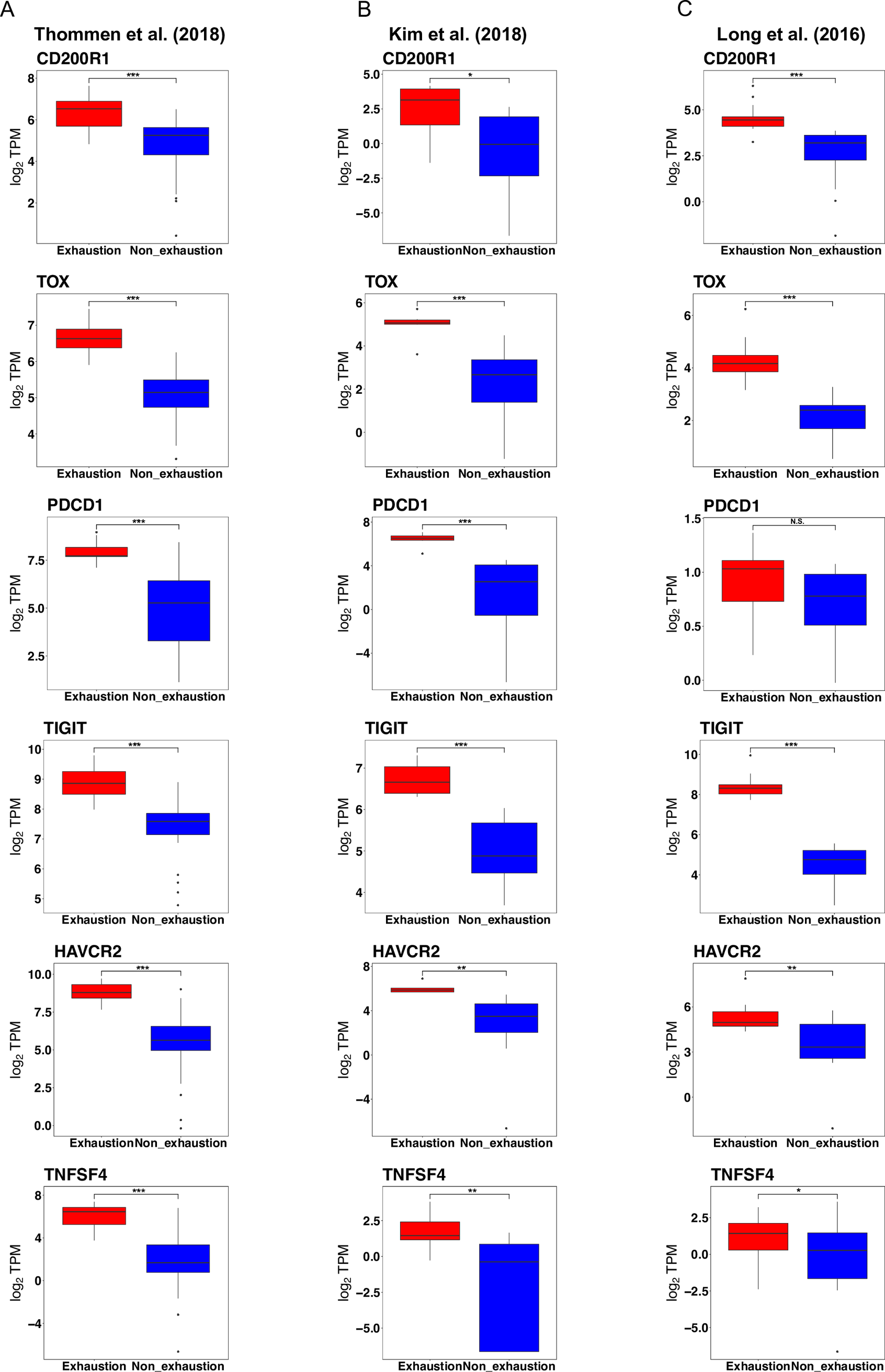

Supplement: S5 Fig — Median values are indicated by lines in the box and whisker plot. Hinge values and whisker 1.5* interquartile range (IQR) values have been calculated. *P < 0.05, ** P < 0.01, *** P < 0.001. (TIF) [file pone.0274494.s005.tif]

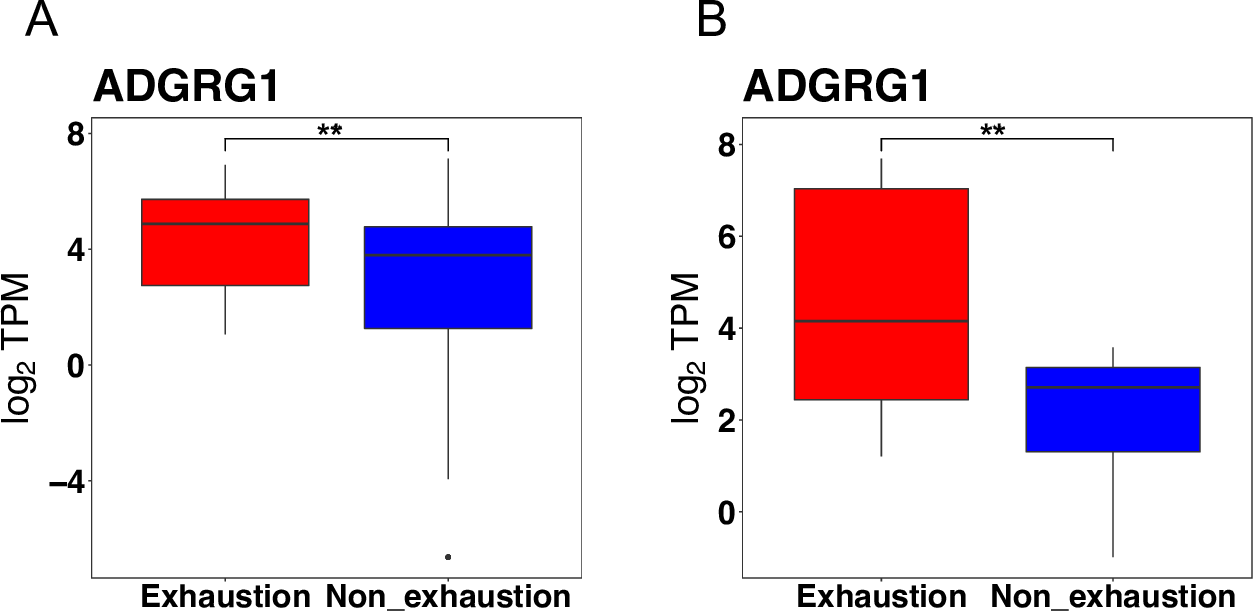

Supplement: S6 Fig — (A) and (B) show the expression of ADGRG1 at the mRNA level in human and mouse datasets, respectively. *P < 0.05, ** P < 0.01, *** P < 0.001. (TIF) [file pone.0274494.s006.tif]
