# Supplementary material for: Transcriptome analysis of Homo sapiens and Mus musculus reveals mechanisms of CD8+ T cell exhaustion caused by different factors
Source: PLoS One. 2022 Sep 9;17(9):e0274494. doi: 10.1371/journal.pone.0274494 (PMC9462770; doi:10.1371/journal.pone.0274494)
Supplement: S1 Table — (DOCX) [file pone.0274494.s007.docx]

**S1 Table. The RNA-seq datasets for *Homo sapiens* and *Mus musculus*.**

| **Species** | **Accession Number** | **Sample Name** | **Title** |
| --- | --- | --- | --- |
| *Homo sapiens* | GSE99531 | GSM2645545 | PD1-high CD8+ T cell [BS-483] |
|  |  | GSM2645546 | PD1-int CD8+ T cell [BS-483] |
|  |  | GSM2645547 | PD1-neg CD8+ T cell [BS-483] |
|  |  | GSM2645548 | PD1-high CD8+ T cell [BS-485] |
|  |  | GSM2645549 | PD1-int CD8+ T cell [BS-485] |
|  |  | GSM2645550 | PD1-neg CD8+ T cell [BS-485] |
|  |  | GSM2645551 | PD1-high CD8+ T cell [BS-278] |
|  |  | GSM2645552 | PD1-int CD8+ T cell [BS-278] |
|  |  | GSM2645553 | PD1-neg CD8+ T cell [BS-278] |
|  |  | GSM2645554 | PD1-high CD8+ T cell [BS-474] |
|  |  | GSM2645555 | PD1-int CD8+ T cell [BS-474] |
|  |  | GSM2645556 | PD1-neg CD8+ T cell [BS-474] |
|  |  | GSM2645557 | PD1-high CD8+ T cell [BS-451] |
|  |  | GSM2645558 | PD1-int CD8+ T cell [BS-451] |
|  |  | GSM2645559 | PD1-neg CD8+ T cell [BS-451] |
|  |  | GSM2645560 | PD1-high CD8+ T cell [BS-358] |
|  |  | GSM2645561 | PD1-int CD8+ T cell [BS-358] |
|  |  | GSM2645562 | PD1-neg CD8+ T cell [BS-358] |
|  |  | GSM2645563 | PD1-high CD8+ T cell [BS-433] |
|  |  | GSM2645564 | PD1-int CD8+ T cell [BS-433] |
|  |  | GSM2645565 | PD1-neg CD8+ T cell [BS-433] |
|  |  | GSM2645566 | PD1-high CD8+ T cell [BS-488] |
|  |  | GSM2645567 | PD1-int CD8+ T cell [BS-488] |
|  |  | GSM2645568 | PD1-neg CD8+ T cell [BS-488] |
|  |  | GSM2645569 | PD1-high CD8+ T cell [BS-200] |
|  |  | GSM2645570 | PD1-int CD8+ T cell [BS-200] |
|  |  | GSM2645571 | PD1-neg CD8+ T cell [BS-200] |
|  |  | GSM2645572 | PD1-high CD8+ T cell [BS-275] |
|  |  | GSM2645573 | PD1-int CD8+ T cell [BS-275] |
|  |  | GSM2645574 | PD1-neg CD8+ T cell [BS-275] |
|  |  | GSM2645575 | PD1-high CD8+ T cell [BS-423] |
|  |  | GSM2645576 | PD1-int CD8+ T cell [BS-423] |
|  |  | GSM2645577 | PD1-neg CD8+ T cell [BS-423] |
|  |  | GSM2645578 | effectory memory T cell [HD-37] |
|  |  | GSM2645579 | effectory memory T cell [HD-55] |
|  |  | GSM2645580 | effectory memory T cell [HD-47] |
|  |  | GSM2645581 | effectory memory T cell [HD-50] |
|  | GSE111389 | GSM3029902 | 31_PD1-high_RNA-Seq |
|  |  | GSM3029903 | 31_PD1-intermediate_RNA-Seq |
|  |  | GSM3029904 | 31_PD1-negative_RNA-Seq |
|  |  | GSM3029905 | 41_PD1-high_RNA-Seq |
|  |  | GSM3029906 | 41_PD1-intermediate_RNA-Seq |
|  |  | GSM3029907 | 41_PD1-negative_RNA-Seq |
|  |  | GSM3029908 | 48_PD1-high_RNA-Seq |
|  |  | GSM3029909 | 48_PD1-intermediate_RNA-Seq |
|  |  | GSM3029910 | 48_PD1-negative_RNA-Seq |
|  |  | GSM3029911 | 52_PD1-high_RNA-Seq |
|  |  | GSM3029912 | 52_PD1-intermediate_RNA-Seq |
|  |  | GSM3029913 | 52_PD1-negative_RNA-Seq |
|  |  | GSM3029914 | 64_PD1-high_RNA-Seq |
|  |  | GSM3029915 | 64_PD1-intermediate_RNA-Seq |
|  |  | GSM3029916 | 64_PD1-negative_RNA-Seq |
|  |  | GSM3029917 | 69_PD1-high_RNA-Seq |
|  |  | GSM3029918 | 69_PD1-intermediate_RNA-Seq |
|  |  | GSM3029919 | 69_PD1-negative_RNA-Seq |
|  | GSE85530 | GSM2274923 | S32683_106306_hOKT3_R_6mo_CD8_TIGITpos_KLRG1pos_stim_untr |
|  |  | GSM2274925 | S32685_106306_hOKT3_R_6mo_CD8_TIGITneg_KLRG1neg_stim_untr |
|  |  | GSM2274927 | S32687_106306_hOKT3_R_6mo_CD8_TIGITpos_KLRG1pos_stim_untr |
|  |  | GSM2274929 | S32689_106306_hOKT3_R_6mo_CD8_TIGITpos_KLRG1pos_stim_untr |
|  |  | GSM2274932 | S32692_106306_hOKT3_R_6mo_CD8_TIGITneg_KLRG1neg_stim_tr |
|  |  | GSM2274937 | S32697_106306_hOKT3_R_6mo_CD8_TIGITneg_KLRG1neg_stim_untr |
|  |  | GSM2274939 | S32722_106306_hOKT3_R_6mo_CD8_TIGITpos_KLRG1pos_stim_untr |
|  |  | GSM2274940 | S32723_106306_hOKT3_R_6mo_CD8_TIGITneg_KLRG1neg_stim_untr |
|  |  | GSM2274941 | S32724_106306_hOKT3_R_6mo_CD8_TIGITpos_KLRG1pos_stim_untr |
|  |  | GSM2274942 | S32725_106306_hOKT3_R_6mo_CD8_TIGITneg_KLRG1neg_stim_untr |
|  |  | GSM2274943 | S32726_106306_hOKT3_R_6mo_CD8_TIGITpos_KLRG1pos_stim_untr |
|  |  | GSM2274945 | S32728_106306_hOKT3_R_6mo_CD8_TIGITneg_KLRG1neg_stim_tr |
|  |  | GSM2274948 | S32731_106306_hOKT3_R_6mo_CD8_TIGITneg_KLRG1neg_stim_tr |
|  |  | GSM2274949 | S33276_838213_hOKT3_R_6mo_CD8_TIGITpos_KLRG1pos_stim_untr |
|  |  | GSM2274950 | S33277_838213_hOKT3_R_6mo_CD8_TIGITneg_KLRG1neg_stim_untr |
|  |  | GSM2274951 | S33278_838213_hOKT3_R_6mo_CD8_TIGITpos_KLRG1pos_stim_untr |
|  |  | GSM2274952 | S33279_838213_hOKT3_R_6mo_CD8_TIGITneg_KLRG1neg_stim_untr |
|  |  | GSM2274953 | S33280_838213_hOKT3_R_6mo_CD8_TIGITpos_KLRG1pos_stim_untr |
|  |  | GSM2274954 | S33281_838213_hOKT3_R_6mo_CD8_TIGITneg_KLRG1neg_stim_untr |
|  |  | GSM2274955 | S33282_838213_hOKT3_R_6mo_CD8_TIGITpos_KLRG1pos_stim_untr |
|  |  | GSM2274956 | S33283_838213_hOKT3_R_6mo_CD8_TIGITneg_KLRG1neg_stim_untr |
|  |  | GSM2274957 | S33284_838213_hOKT3_R_6mo_CD8_TIGITpos_KLRG1pos_stim_untr |
|  |  | GSM2274958 | S33285_838213_hOKT3_R_6mo_CD8_TIGITneg_KLRG1neg_stim_tr |
|  |  | GSM2274960 | S33287_838213_hOKT3_R_6mo_CD8_TIGITneg_KLRG1neg_stim_tr |
|  |  | GSM2274964 | S33347_618372_hOKT3_R_6mo_CD8_TIGITpos_KLRG1pos_stim_untr |
|  |  | GSM2274965 | S33348_618372_hOKT3_R_6mo_CD8_TIGITneg_KLRG1neg_stim_untr |
|  |  | GSM2274966 | S33349_618372_hOKT3_R_6mo_CD8_TIGITpos_KLRG1pos_stim_untr |
|  |  | GSM2274967 | S33350_618372_hOKT3_R_6mo_CD8_TIGITneg_KLRG1neg_stim_untr |
|  |  | GSM2274968 | S33351_618372_hOKT3_R_6mo_CD8_TIGITpos_KLRG1pos_stim_untr |
|  |  | GSM2274969 | S33352_618372_hOKT3_R_6mo_CD8_TIGITneg_KLRG1neg_stim_tr |
|  |  | GSM2274970 | S33353_618372_hOKT3_R_6mo_CD8_TIGITpos_KLRG1pos_stim_untr |
|  |  | GSM2274971 | S33354_618372_hOKT3_R_6mo_CD8_TIGITneg_KLRG1neg_stim_tr |
|  |  | GSM2274972 | S33355_618372_hOKT3_R_6mo_CD8_TIGITpos_KLRG1pos_stim_untr |
| *Mus musculus* | GSE93006 | GSM2442410 | OTI_r1 |
|  |  | GSM2442410 | OTI_r1 |
|  |  | GSM2442410 | OTI_r1 |
|  |  | GSM2442410 | OTI_r1 |
|  |  | GSM2442411 | OTI_r2 |
|  |  | GSM2442412 | OTI_r3 |
|  |  | GSM2442413 | P14_r1 |
|  |  | GSM2442413 | P14_r1 |
|  |  | GSM2442413 | P14_r1 |
|  |  | GSM2442413 | P14_r1 |
|  |  | GSM2442414 | P14_r2 |
|  |  | GSM2442414 | P14_r2 |
|  |  | GSM2442414 | P14_r2 |
|  |  | GSM2442414 | P14_r2 |
|  |  | GSM2442415 | P14_r3 |
|  |  | GSM2442415 | P14_r3 |
|  |  | GSM2442416 | P14_r4 |
|  |  | GSM2442417 | P14_r5 |
|  | GSE84820 | GSM2251840 | WT Acute d30 REP1 |
|  |  | GSM2251848 | WT Acute d30 REP2 |
|  |  | GSM2251851 | WT Acute d30 REP3 |
|  |  | GSM2251872 | WT Acute d30 REP4 |
|  |  | GSM2251845 | WT Chronic d30 REP1 |
|  |  | GSM2251850 | WT Chronic d30 REP2 |
|  | GSE83978 | GSM2224778 | Donnor2 d28 Memory Tcf1+ |
|  |  | GSM2224780 | Donnor2 d28 Chronic Tcf1+ |
|  |  | GSM2224783 | Donnor3 d28 Memory Tcf1+ |
|  |  | GSM2224785 | Donnor1 d28 Chronic Tcf1+ |
|  |  | GSM2224787 | Donnor4 d28 Memory Tcf1+ |
|  |  | GSM2224789 | Donnor4 d28 Chronic Tcf1+ |
|  | GSE86881 | GSM2309812 | RNA-seq of CD8 T cells from spleen-exhausted_untreated, 1 |
|  |  | GSM2309813 | RNA-seq of CD8 T cells from spleen-exhausted_untreated, 2 |
|  |  | GSM2309814 | RNA-seq of CD8 T cells from spleen-exhausted_untreated, 3 |
|  |  | GSM2309819 | RNA-seq of CD8 T cells from spleen-exhausted_untreated-day235 |
|  |  | GSM2309820 | RNA-seq of CD8 T cells from spleen-exhausted_untreated-day160 |
