# Supplementary material for: Transcriptome analysis of Homo sapiens and Mus musculus reveals mechanisms of CD8+ T cell exhaustion caused by different factors
Source: PLoS One. 2022 Sep 9;17(9):e0274494. doi: 10.1371/journal.pone.0274494 (PMC9462770; doi:10.1371/journal.pone.0274494)
Supplement: S2 Table — (DOCX) [file pone.0274494.s008.docx]

**S2 Table. DEGs and their matched genes for each enriched process and pathway in Fig 2 and S2 Fig.**

| ***Homo sapiens*** |  |  |
| --- | --- | --- |
| DEGs | Upregulation | NDFIP2, MT1L, MYO1E, RRM2, CTLA4, AC022506.1, BHLHE40-AS1, TOX, AFAP1, PKMYT1, MAP1LC3A, MAMLD1, BATF3, RN7SL743P, TRAJ13, TNIP3, RNU6-786P, MIR3142HG, AC009088.2, HLA-DMA, MSC, AIMP2, ASB2, ADGRG1, AC023794.3, IGLV2-14, AC090152.1, TRBV27, TRAJ54, ENO1P1, NMB, LDHAP2, CCR5, MIR7848, AC145207.3, AL590133.2, RPL17-C18orf32, MIR155HG, ST8SIA1, FAM184A, AC078927.1, BUB1B, RAD51AP1, TIAM2, AKAP5, TRAJ22, AC008982.1, TRAJ42, EPOP, RNU6-1016P, C3orf14, CD200R1, TOX2, CCL4L2, FAM111B, IKZF4, RF02195, SETBP1, TRAJ32, RF00017, SNORD36A, AC115102.1, FCRL3, SLC5A2, INPP5F, AP005263.1, LINC01480, AL022067.1, NCS1, AC133644.2, AC120057.2, SLC27A2, CLECL1, BLOC1S5-TXNDC5, SECTM1, HIST1H2AJ, BCAT1, ENTPD1, RNY3P14, MIR4426, CCL3L1, AL928654.2, FAM3C2, RF00019, AL512625.3, AC006111.1, RNU6-645P, CDCA3, MT-TG, MT-TL2, RF00019, RAMP1, AL157871.4, SNORA61, TRGJP2, SNORA77, MELK, TNFRSF9, ADAM28, DTHD1, AC080023.2, SLC2A8, AL355816.1, LAIR2, AL354798.1, PTGES3P2, RDH10, SNORA70, RNU6-268P, AL360012.1, ST20-MTHFS, PDCD1, AC099329.1, AC009093.8, RN7SL653P, AC092069.1, RF02196, AC011247.2, CADM1, PDGFA, TRBV7-9, PIF1, RF00019, MSC-AS1, MT-TR, RF00019, RF00413, AL158071.2, CHN1, RN7SL559P, HAVCR2, KRT86, RN7SL246P, CCL3, RF00019, MS4A6A, RNY4P34, RNU7-1, AC009244.1, MRPS36P1, RAB38, CD86, TIGIT, CAMK1, ETV1, HECTD2, TRAJ6, TNFSF4, AC020978.5, AC090844.1, MIR6764, AC073261.1, SCARNA22, AC006064.4, LINC00271, TRAJ52, AC027319.1, KIR2DL4, RF00019, DRAIC, TRAJ4, VCAM1, AC009656.1, MYO7A, ATP8B4, CD200, LMCD1, AC013460.1, TIMD4, LINC01943, MIR4647, ZBTB20-AS1, RF02105, AC017002.3, AL035604.1, HMOX1, GNG4, AC145207.2, LINC00515, IL13, MT-TK, ZBED2, TMEM155, AL158071.3, TRAJ3, SNORD15B, RF00397, AC243829.4, SNORD104, GLDC, LINC02341, SNORD11, AC090498.1, HMGB1P17, PMCH, FNDC9, PPIAP45, MIR155, CXCL13 |
|  | Downregulation | MYH10, TNFSF8, SERPINI1, FBXL14, FTH1P2, MGAT4A, FAM157B, NDRG2, SPATC1L, PBXIP1, IL1B, IGLV3-1, RASGRP3, SNN, AC008555.5, GZMM, IL4I1, AC108449.2, TSGA10, TRAV25, MIR6516, ZFP36L2, TRAV1-1, ETFRF1, IGLV1-47, HSPA7, GPR132, GLUL, RASA3, TMSB4XP4, DECR2, DUSP6, AL136454.1, AL137779.2, FTH1, ZBTB10, SNORD107, IGHV3-11, IGKV1-5, PIK3R1, STX3, FOS, TRBV20-1, LIPT2, SNAI3, AC127502.2, MYADM, CITED4, SNORD49B, AEBP1, GSAP, CXCR4, AC005912.1, KIFC2, AC020656.1, SMIM14, AHNAK, NME4, WFDC2, MYBL1, ANTXR2, AL590867.2, RARA, HIST4H4, SNORD32A, NEIL1, LGALS3BP, AL162377.1, RF02271, RNU6-890P, CCL2, IGFBP4, PERP, ARHGAP5, TRDC, RPL13AP7, TMEM63A, AL671277.1, LTB, DUSP1, GALNT6, TF, TRAF4, RF00004, SNORA12, MGLL, STAG3, RNU6-387P, AC021188.1, RNVU1-7, IRF2BPL, AC095055.1, TC2N, SCML1, TRAV36DV7, P2RY8, CKAP4, RAB11FIP5, HIST1H2BC, IGLV3-9, MT1G, AC105020.2, ARL4D, LINC002481, TM4SF1, HSPA2, AL671762.1, IGHV1-18, IGKV3-11, SNORD21, IGHV4-39, CX3CR1, FHL1, FGL2, PKIG, TMEM71, GZMK, SNORD20, RPS7P11, ENC1, IL22, PFN2, AC004585.1, TRABD2A, GPR183, MARCH3, BACH2, CST3, PLEKHB1, IGHV3-23, ANXA1, SVIL, DDIT4, AC099336.2, CXCL1, RNF166, IGKV2D-28, IGLV1-40, ATP1B1, IGHD, ORM1, THBS1, TSC22D3, RF00019, SMIM3, IGLV3-25, ANAPC2, RF00003, GNLY, ZSCAN18, SCARNA4, PPP1R2C, TRAV10, EMP1, PXN, CCR4, DUSP8, TSPAN2, TRBV5-5, SLC16A3, BCL9L, FGR, SNORD33, HIST1H2BD, IGKV2-28, DNAJB1, MAFF, GPR35, HMGN5, LMNA, IL3, CCR7, SNORD59A, FCER1G, GNG8, PTGDR, AC020765.2, AL365357.1, PDCD4-AS1, ACSL6, RF02166, RAB13, AC013264.1, IL12RB2, TRGV10, EPHA1-AS1, CD40LG, CXCL8, TUFT1, RPS20P14, PSMD6-AS2, GALNT3, RPL10P6, IGFBP7, CYCSP45, F8A3, NCF1, HRH2, TGFBI, LYZ, ZMAT1, COL1A2, ADRB2, TNFSF13B, SNORD13E, NUDT4P2, USP53, RPL34P27, DKK3, LDLRAP1, HIST1H2AC, SNORA10B, SERPING1, ULBP2, AL592183.1, AL591895.1, KRT19, SNORD23, MS4A1, GTSF1, SNORD55, IGKV3-20, AC109326.1, CFB, LINC02384, C11orf21, EFNA1, AC113935.1, RPS27P29, MIR616, KRT8, HIST2H2BE, AL121845.2, KRT18, TMEM176B, AC020571.1, CEBPD, SNORA26, CXCL3, MPZL2, IGHV1-3, MT1XP1, IGLV3-21, SPARC, EPHA1, AL928654.3, TNFAIP2, AL591846.1, UBE2E2, SNORD3B-1, YWHAZP5, CXCL2, SERPINA1, SYNGR1, AC004918.1, BEX5, AC026401.2, RF00003, CTH, ITGA5, RNU1-134P, LSR, RF01979, FCRL6, SNORD12C, RPL21P119, DHRS3, TRAV21, TRBV4-2, SNORA7A, AC006064.5, IGHV1-2, TXK, SPINT2, C9orf72, SNORA84, RMRP, TRBV10-2, SIK1B, AGPAT4, SFTPB, ITGA6, SLCO3A1, TSPAN32, WDR86-AS1, LARGE2, IGLC3, SERPINF1, GDPD5, LST1, AL627402.1, S1PR5, MMP9, RF00019, IL7R, KLF2, RNU1-120P, AC124312.5, FAM169A, IGHG3, SNORD94, CXCL16, AC127502.1, RPL10AP2, HIST1H2BG, IGLC2, SNORA2C, AC099560.2, SNORD14D, SNORD45A, SNORA11F, RNF144A, PLAUR, ZNF165, S100A9, CD300A, CLU, FTH1P10, MIR142, SBF2, DPP4, PCAT1, JCHAIN, SNORD22, ZBTB16, EEF1B2P6, TRAV19, MYOM2, MGP, FTH1P20, CES1, LGMN, AC011497.2, AC135068.8, SPP1, IGHV1OR15-1, TCF7, RNU6-476P, AC004832.6, APBA2, SEMA4C, IFNGR2, CCDC65, SNORD35A, AUTS2, FGF9, TRBV4-1, RNA5SP371, SPON2, RPL30P4, SNORD89, TRBV24-1, CALHM6, RPL34P18, NHSL2, AL121944.1, MPP7, LAIR1, LYPD3, SNORD91A, PLCB1, TRBV6-1, GFPT2, SORL1, SSBP2, TYROBP, PLK2, C3AR1, COL6A2, RF00003, IRS2, ISCA1P1, C1QB, LEF1, BCYRN1, C1QA, SORBS3, RASGRP2, EPHA4, KLF3, TLE1, DPEP2, AC093591.1, IGKC, GPR15, FTH1P7, HIST2H2AA4, S1PR1, IGFBP3, AL161787.1, RN7SL34P, CRIP2, LINC00239, CYB561, IL2, RF00015, AL109741.1, MIR6891, TRBV15, RPS29P5, RPL39P3, TIMP1, IGHG4, TRBV12-5, CNN3, GRASP, FGFBP2, HIST2H2AA3, CACNA1C-AS2, G0S2, IGHA1, TRBV6-4, C1orf162, TRBV7-3, NSG1, AL162151.2, TRBV10-3, RNVU1-14, LINC02273, TMIGD2, SPART, IGHG1, RF00283, KLRF1, AQP3, RF00019, TRBV25-1, VTRNA1-3, AC016596.1, MT-TQ, PLEKHG3, PLAC8, ZNF683, PLXDC1, MIR663A, CCR6, MIR1244-1, MIR1244-4, MIR1244-2, RPL41P5, RPL41P1 |
| GO_upregulation | Regulation of response to stimulus | AFAP1, AKAP5, CADM1, CCL3, CCL3L3, CCL4L1, CD200, CD200R1, CD86, CHN1, CTLA4, CXCL13, FCRL3, GNG4, GPR56, HAVCR2, HMOX1, IL13, INPP5F, KIR2DL4, LAIR2, LMCD1, NMB, PDGFA, PMCH, RAD51AP1, RAMP1, SECTM1, TIAM2, TNFSF4, TNIP3, VCAM1 |
|  | Immune system process | ATP8B4, BATF3, CADM1, CCL3, CCL3L3, CCL4L1, CCR5, CD86, CTLA4, CXCL13, HAVCR2, HLA-DMA, HMOX1, IL13, MELK, MYO1E, PDCD1, SECTM1, SLC27A2, TNFRSF9, TNFSF4, TNIP3, TOX, VCAM1 |
|  | Inflammatory response | CCL3, CCL3L3, CCL4L1, CCR5, CXCL13, HAVCR2, HMOX1, IL13, TNFRSF9, TNFSF4, TNIP3, VCAM1 |
|  | Cytokine-mediated signaling pathway | CCL3, CCL3L3, CCL4L1, CCR5, CD86, CXCL13, HMOX1, IL13, TNFRSF9, TNFSF4, VCAM1 |
|  | Response to lipid | CCL3, CCR5, CXCL13, GLDC, HAVCR2, IL13, TNFRSF9, TNFSF4, TNIP3, VCAM1, YY1 |
|  | Response to lipopolysaccharide | CCL3, CCR5, CXCL13, HAVCR2, IL13, TNFRSF9, TNFSF4, TNIP3, VCAM1 |
|  | Negative regulation of immune system process | CCL3, CD200, CTLA4, FCRL3, HAVCR2, HMOX1, PDCD1, TIGIT, TNFSF4 |
|  | Regulation of T cell activation | CD86, CLECL1, CTLA4, HAVCR2, PDCD1, TIGIT, TNFSF4, VCAM1 |
|  | Negative regulation of cytokine production | HAVCR2, HMOX1, IL13, TIGIT, TNFRSF9, TNFSF4, YY1 |
|  | Regulation of lymphocyte proliferation | CLECL1, CTLA4, FCRL3, HAVCR2, IL13, TNFSF4, VCAM1 |
|  | Cellular response to tumor necrosis factor | CCL3, CCL3L3, CCL4L1, TNFRSF9, TNFSF4, VCAM1 |
|  | Regulation of leukocyte differentiation | CCL3, CD86, CTLA4, FCRL3, TNFSF4, TOX |
|  | Leukocyte chemotaxis | CCL3, CCL3L3, CCL4L1, CCR5, CXCL13 |
|  | T cell costimulation | CD86, CTLA4, PDCD1 |
|  | Negative regulation of regulatory T cell differentiation | CTLA4, TNFSF4 |
| GO_downregulation | Regulation of cellular process | ADRB2, AEBP1, AHNAK, ANAPC2, ANTXR2, ANXA1, AQP3, ARHGAP5, ATP1B1, AUTS2, BACH2, BCL9L, C3AR1, C9orf72, CCDC65, CCL2, CCR4, CCR7, CD300A, CD40LG, CEBPD, CITED4, CLU, COL1A2, CRIP2, CST3, CTH, CX3CR1, CXCL1, CXCL16, CXCL2, CXCL3, CXCL8, CXCR4, DDIT4, DHRS3, DKK3, DNAJB1, DPP4, DUSP1, DUSP6, DUSP8, EFNA1, ENC1, EPHA1, EPHA4, FCER1G, FGF9, FGR, FHL1, FOS, FTH1, G0S2, GALNT3, GDPD5, GLUL, GNG8, GPR132, GPR15, GPR183, GPR29, GPR35, GRASP, GSAP, HIST1H2AC, HIST2H2AA, HIST2H2AA3, HIST4H4, HMGN5, HRH2, HSPA2, IFNGR2, IGFBP3, IGFBP4, IGFBP7, IGHV3-11, IGJ, IL12RB2, IL1B, IL2, IL22, IL3, IL7R, IRF2BPL, IRS2, ITGA5, ITGA6, KLF2, KLF3, KLRF1, KRT18, KRT19, KRT8, LDLRAP1, LEF1, LGALS3BP, LGMN, LIPT2, LMNA, LST1, LTB, MAFF, MGLL, MMP9, MPP7, MYADM, MYBL1, MYH10, NCF1, NDRG2, NEIL1, NME4, NSG1, ORM1, P2RY8, PBXIP1, PERP, PFN2, PIK3R1, PKIG, PLAC8, PLAUR, PLCB1, PLEKHB1, PLEKHG3, PLK2, PTGDR, PXN, RAB11FIP5, RAB13, RARA, RASA3, RASGRP2, RASGRP3, RNF144A, RNF166, S100A9, S1PR1, S1PR5, SCML1, SEMA4C, SERPINA1, SERPINF1, SERPING1, SERPINI1, SNAI3, SORBS3, SORL1, SPARC, SPG20, SPINT2, SPP1, SSBP2, STX3, SVIL, SYNGR1, TCF7, TF, TGFBI, THBS1, TIMP1, TLE1, TMEM176B, TMIGD2, TNFSF13B, TNFSF8, TRABD2A, TRAF4, TSC22D3, TSPAN2, TSPAN32, TXK, TYROBP, UBE2E2, WFDC2, ZBTB10, ZBTB16, ZFP36L2, ZMAT1, ZNF165, ZNF683, ZSCAN18, hCG_401294 |
|  | Regulation of metabolic process | ADRB2, AEBP1, AHNAK, ANXA1, APBA2, ATP1B1, AUTS2, BACH2, BCL9L, C1QA, C1QB, C3AR1, C9orf72, CCL2, CCR7, CD300A, CD40LG, CEBPD, CFB, CITED4, CLU, CST3, CTH, CXCR4, DDIT4, DKK3, DNAJB1, DUSP1, DUSP6, DUSP8, EFNA1, ENC1, EPHA1, EPHA4, FGF9, FGR, FOS, G0S2, GPR183, GSAP, HIST1H2AC, HIST2H2AA, HIST2H2AA3, HIST4H4, HMGN5, HSPA2, IGFBP3, IGFBP4, IGFBP7, IGHV3-11, IGJ, IL1B, IL2, IL3, IL7R, IRF2BPL, IRS2, ITGA5, ITGA6, KLF2, KLF3, LDLRAP1, LEF1, LGMN, LIPT2, LMNA, LSR, LTB, MAFF, MMP9, MYADM, MYBL1, NCF1, NDRG2, NEIL1, NSG1, PBXIP1, PERP, PFN2, PIK3R1, PKIG, PLAC8, PLAUR, PLCB1, PLK2, RARA, RNF144A, RNF166, S100A9, S1PR1, SCML1, SEMA4C, SERPINA1, SERPINF1, SERPING1, SERPINI1, SNAI3, SORBS3, SORL1, SPG20, SPINT2, SPP1, SSBP2, TCF7, THBS1, TIMP1, TLE1, TNFSF8, TRABD2A, TRAF4, TSC22D3, TXK, WFDC2, ZBTB10, ZBTB16, ZFP36L2, ZNF165, ZNF683, ZSCAN18 |
|  | Signaling | ADRB2, ANTXR2, ANXA1, APBA2, ARHGAP5, ATP1B1, BCL9L, C1QA, C3AR1, CCL2, CCR4, CCR7, CD300A, CD40LG, CEBPD, CLU, COL1A2, CTH, CX3CR1, CXCL1, CXCL16, CXCL2, CXCL3, CXCL8, CXCR4, DDIT4, DKK3, DUSP1, DUSP6, EFNA1, EPHA1, EPHA4, FCER1G, FGF9, FGR, FOS, G0S2, GALNT3, GNG8, GPR132, GPR15, GPR183, GPR29, GPR35, GRASP, HRH2, IFNGR2, IGFBP4, IGHV3-11, IL12RB2, IL1B, IL2, IL22, IL3, IL7R, IRS2, ITGA5, ITGA6, KLRF1, KRT18, KRT19, KRT8, LEF1, LGALS3BP, LMNA, LTB, MMP9, NCF1, NDRG2, NSG1, P2RY8, PERP, PIK3R1, PKIG, PLAUR, PLCB1, PLEKHB1, PLK2, PTGDR, PXN, RAB11FIP5, RAB13, RARA, RASA3, RASGRP2, RASGRP3, S100A9, S1PR1, S1PR5, SEMA4C, SORL1, STX3, TCF7, TIMP1, TLE1, TMIGD2, TNFSF13B, TNFSF8, TRABD2A, TRAF4, TSPAN2, TSPAN32, TXK, TYROBP, ZFP36L2, ZMAT1, ZNF683, hCG_401294 |
|  | System development | AEBP1, ANAPC2, ANXA1, APBA2, AQP3, ARHGAP5, AUTS2, BCL9L, C1QB, C9orf72, CCL2, CCR4, CCR7, CD40LG, CLU, COL1A2, CRIP2, CST3, CX3CR1, CXCL1, CXCL8, CXCR4, DDIT4, DHRS3, DKK3, DNAJB1, EFNA1, ENC1, EPHA1, EPHA4, FCER1G, FGF9, FHL1, FOS, GDPD5, GNG8, GPR183, GPR29, HRH2, IGFBP4, IL2, IL3, IL7R, IRS2, ITGA5, ITGA6, KLF2, KRT18, KRT19, KRT8, LEF1, LMNA, LSR, LST1, LTB, MAFF, MGP, MMP9, MT1G, MYH10, MYOM2, PERP, PIK3R1, PLCB1, PLK2, PLXDC1, RAB13, RARA, S100A9, S1PR1, S1PR5, SBF2, SEMA4C, SERPINF1, SERPINI1, SFTPB, SORL1, SPARC, SPG20, SPINT2, SPON2, SPP1, STX3, SVIL, TCF7, TGFBI, THBS1, TIMP1, TLE1, TMEM176B, TNFAIP2, TNFSF8, TRAF4, TSPAN2, TUFT1, TYROBP, ZBTB16, ZFP36L2 |
|  | Localization | ADRB2, ANTXR2, ANXA1, APBA2, AQP3, ARL4D, ATP1B1, AUTS2, BACH2, C3AR1, C9orf72, CCL2, CCR4, CCR7, CD300A, CD40LG, CES1, CKAP4, CLU, COL1A2, CST3, CX3CR1, CXCL1, CXCL16, CXCL2, CXCL3, CXCL8, CXCR4, DDIT4, DECR2, DPP4, EFNA1, EPHA4, FAM26F, FCER1G, FGF9, FGL2, FGR, FTH1, GLUL, GPR15, GPR183, GPR29, GRASP, HRH2, IGHV3-11, IGJ, IL1B, ITGA5, ITGA6, KRT18, LAIR1, LDLRAP1, LEF1, LGALS3BP, LMNA, LSR, LYZ, MARCH3, MMP9, MPP7, MYADM, MYH10, NCF1, NME4, NSG1, ORM1, PIK3R1, PLAC8, PLAUR, PXN, RAB11FIP5, RAB13, RARA, RASA3, S100A9, S1PR1, SEMA4C, SERPINA1, SERPING1, SLC16A3, SLCO3A1, SORL1, SPARC, SPON2, STX3, SYNGR1, TF, THBS1, TIMP1, TMEM63A, TNFAIP2, TNFSF13B, TSC22D3, TYROBP, ZBTB16 |
|  | Regulation of molecular function | ADRB2, AHNAK, ANXA1, ARHGAP5, ATP1B1, C9orf72, CCL2, CCR7, CD300A, CD40LG, CLU, CNN3, CST3, CTH, CXCL1, CXCL16, CXCL2, CXCL3, CXCL8, CXCR4, DNAJB1, DUSP1, DUSP6, DUSP8, EFNA1, EPHA1, EPHA4, FGF9, FGR, FHL1, FOS, GPR35, HSPA2, IGFBP3, IL1B, IL2, IL22, IL3, IRS2, ITGA6, LDLRAP1, LEF1, LGMN, LTB, MMP9, NCF1, NEIL1, PERP, PFN2, PIK3R1, PKIG, PLAUR, PLCB1, PLEKHG3, PLK2, RARA, RASA3, RASGRP2, RASGRP3, S100A9, S1PR1, SBF2, SERPINA1, SERPINF1, SERPING1, SERPINI1, SFTPB, SORL1, SPINT2, SPP1, THBS1, TIMP1, TNFSF13B, TNFSF8, TRAF4, TXK, WFDC2 |
|  | Positive regulation of response to stimulus | ADRB2, ANXA1, AUTS2, C1QA, C1QB, C3AR1, CCL2, CCR4, CCR7, CD40LG, CFB, CLU, CTH, CXCL1, CXCL2, CXCL3, CXCL8, CXCR4, DUSP6, EFNA1, EPHA4, FCER1G, FGF9, FGR, G0S2, GPR183, GPR29, GPR35, IGFBP3, IGFBP4, IGHV3-11, IL1B, IL2, IL3, IL7R, IRS2, ITGA5, LDLRAP1, LGMN, MMP9, MPP7, NCF1, P2RY8, PIK3R1, PLAUR, PLCB1, RARA, S100A9, S1PR1, SEMA4C, SERPING1, SORBS3, SPON2, STX3, THBS1, TNFSF13B, TRAF4, TXK, ZNF683 |
|  | Macromolecule modification | ADRB2, ANAPC2, ANXA1, BACH2, CCL2, CD40LG, CKAP4, CST3, CTH, DUSP1, DUSP6, DUSP8, ENC1, EPHA1, EPHA4, FBXL14, FGF9, FGR, FOS, GALNT3, GALNT6, GFPT2, GYLTL1B, HIST1H2BC, HIST1H2BD, HIST1H2BG, IGFBP3, IGFBP4, IGFBP7, IL12RB2, IL1B, IL2, IL3, IRS2, LEF1, LIPT2, MARCH3, MGAT4A, NEIL1, PIK3R1, PLK2, RAB13, RARA, RASA3, RASGRP3, RNF144A, RNF166, S100A9, SERPINA1, SPP1, TF, THBS1, TIMP1, TXK, UBE2E2, USP53, ZBTB16, ZFP36L2, hCG_401294 |
|  | Vesicle-mediated transport | ADRB2, ANXA1, C3AR1, C9orf72, CD300A, CES1, CKAP4, CLU, CST3, CXCL1, CXCL16, CXCL8, FCER1G, FGL2, FGR, FTH1, IGHV3-11, IGJ, KRT18, LAIR1, LDLRAP1, LGALS3BP, LYZ, MARCH3, MMP9, MYH10, NSG1, ORM1, PIK3R1, PLAC8, PLAUR, RAB11FIP5, RAB13, RARA, S100A9, SERPINA1, SERPING1, SORL1, SPARC, SPON2, STX3, SYNGR1, TF, THBS1, TIMP1, TMEM63A, TNFAIP2, TYROBP |
|  | Regulation of cell death | ANXA1, CCL2, CCR7, CD40LG, CLU, CST3, CTH, CX3CR1, DDIT4, DUSP1, DUSP6, EFNA1, FCER1G, FOS, G0S2, HMGN5, IGFBP3, IL1B, IL2, IL7R, IRS2, ITGA5, ITGA6, KRT18, LEF1, LGMN, LMNA, MMP9, NME4, PERP, PIK3R1, PLAC8, PLAUR, PLK2, RARA, S100A9, SERPINF1, SORL1, TCF7, THBS1, TIMP1, TLE1, TRAF4, TSC22D3, ZBTB16 |
|  | Regulation of phosphorylation | ADRB2, C9orf72, CCL2, CCR7, CD300A, CD40LG, CLU, CXCR4, DDIT4, DUSP1, DUSP6, DUSP8, EFNA1, EPHA1, EPHA4, FGF9, FGR, GPR183, HSPA2, IGFBP3, IGFBP4, IL1B, IL2, IL3, IRS2, ITGA5, ITGA6, MMP9, MYADM, NCF1, NDRG2, PFN2, PIK3R1, PKIG, PLAUR, PLCB1, SEMA4C, SORBS3, SORL1, THBS1, TRAF4 |
|  | Cell migration | ANXA1, ATP1B1, AUTS2, C3AR1, CCL2, CCR4, CCR7, COL1A2, CX3CR1, CXCL1, CXCL16, CXCL2, CXCL3, CXCL8, CXCR4, DDIT4, DPP4, EFNA1, EPHA4, FCER1G, FGR, GPR15, GPR183, GPR29, IGHV3-11, IGJ, IL1B, ITGA5, ITGA6, LEF1, MMP9, MYH10, PIK3R1, PXN, RAB13, S100A9, S1PR1, SEMA4C, SLC16A3, THBS1 |
|  | Positive regulation of immune system process | ANXA1, AQP3, C1QA, C1QB, C3AR1, CCL2, CCR7, CD300A, CD40LG, CFB, CLU, CXCL1, CXCL2, CXCL3, CXCL8, DPP4, FCER1G, FGR, FOS, GPR183, GPR29, IGHV3-11, IL1B, IL2, IL7R, IRS2, LEF1, LGMN, PIK3R1, RARA, S100A9, SERPING1, SPON2, THBS1, TMIGD2, TNFSF13B, TXK, ZBTB16, ZNF683 |
|  | Cell adhesion | ARHGAP5, ATP1B1, CCL2, CD300A, CD40LG, COL6A2, CX3CR1, DPP4, EFNA1, EPHA1, EPHA4, IGFBP7, IL1B, IL2, ITGA5, ITGA6, LEF1, LGALS3BP, LYPD3, MPZL2, MYH10, PERP, PXN, S100A9, S1PR1, SORBS3, SPON2, SPP1, TGFBI, THBS1, TSPAN32 |
|  | Positive regulation of MAPK cascade | ADRB2, CCL2, CCR7, CD40LG, CXCR4, DUSP6, EFNA1, EPHA4, FGF9, GPR183, IGFBP3, IGFBP4, IL1B, NCF1, PLCB1, SEMA4C, SORBS3, THBS1, TRAF4 |
| KEGG_upregulation | Cytokine-cytokine receptor interaction | CCL3, CCL3L3, CCL4L1, CCR5, CXCL13, IL13, PDGFA, TNFRSF9, TNFSF4 |
|  | Cell adhesion molecules (CAMs) | CADM1, CD86, CTLA4, HLA-DMA, PDCD1, TIGIT, VCAM1 |
|  | Chemokine signaling pathway | CCL3, CCL3L3, CCL4L1, CCR5, CXCL13, GNG4 |
|  | Rheumatoid arthritis | CCL3, CCL3L3, CD86, CTLA4, HLA-DMA |
|  | Toll-like receptor signaling pathway | CCL3, CCL3L3, CCL4L1, CD86 |
|  | Autoimmune thyroid disease | CD86, CTLA4, HLA-DMA |
| KEGG_downregulation | Cytokine-cytokine receptor interaction | CCL2, CCR4, CCR7, CD40LG, CX3CR1, CXCL1, CXCL16, CXCL2, CXCL3, CXCL8, CXCR4, IFNGR2, IL12RB2, IL1B, IL2, IL22, IL3, IL7R, LTB, TNFSF13B, TNFSF8 |
|  | Pathways in cancer | CXCL8, CXCR4, FGF9, FOS, GNG8, IFNGR2, IL12RB2, IL2, IL3, IL7R, ITGA6, LEF1, MMP9, PIK3R1, PLCB1, RARA, RASGRP2, RASGRP3, TCF7, TRAF4, ZBTB16 |
|  | Chemokine signaling pathway | CCL2, CCR4, CCR7, CX3CR1, CXCL1, CXCL16, CXCL2, CXCL3, CXCL8, CXCR4, FGR, GNG8, NCF1, PIK3R1, PLCB1, PXN, RASGRP2 |
|  | IL-17 signaling pathway | CCL2, CXCL1, CXCL2, CXCL3, CXCL8, FOS, IL1B, MMP9, S100A9, TRAF4 |
|  | Chagas disease (American trypanosomiasis) | C1QA, C1QB, CCL2, CXCL8, FOS, IFNGR2, IL1B, IL2, PIK3R1, PLCB1 |
|  | Salmonella infection | CXCL1, CXCL2, CXCL3, CXCL8, FOS, IFNGR2, IL1B, MYH10, PFN2 |
| ***Mus musculus*** |  |  |
| DEGs | Upregulation | ZFP14, MIS18BP1, SIRT5, ZFP937, AC168977.2, CENPU, MOCS3, IFNG, GBP10, NRBF2, GM14419, GM25767, GM20939, GM2436, FCNA, TMEM14A, GM48720, PLOD2, TRAV8-2, GEMIN8, SEC22A, CIP2A, ARHGAP39, ARL10, GM20388, GM28911, TESK2, GM43778, GM14085, GM6741, GLCCI1, STK3, LITAF, TLCD2, GM3055, CCNE2, FEN1, RNF43, ANKRD46, B230354K17RIK, GPD2, IGHV3-6, APOO, ZFP41, NANP, GTSE1, MMD, RPH3AL, STX11, ZFP970, PRNP, MT-TP, TRAIP, GM15542, TOP2A, GM13835, D130040H23RIK, TRMT61B, GEM, MT-TN, HOOK1, EID2, KDELC1, GM47248, KIF18B, MS4A1, IRF8, CLSPN, CDCA8, HELLS, 1700012D14RIK, NRIP1, ALCAM, CEP55, FMNL3, IFI44, DNAJC12, 1110065P20RIK, MT-TC, GM22107, GM4814, 4632427E13RIK, GM29094, TCRG-V1, FAU-PS2, ETAA1OS, MIR6357, MIR546, GM22247, CKAP2L, LNCPINT, UHRF1, GM14681, GM17494, CCNA2, GM26130, PLSCR1, GM44973, IGKV8-24, GM7609, PARD6A, PGM3, SKA3, GPATCH3, CCNF, CENPM, XKRX, CDH23, AC167249.1, GM25744, MRNIP, CSF1, DECR1, AC153498.1, 2810408I11RIK, ABHD4, MTRF1, MARCKSL1, CD160, GM38329, CD38, CENPP, GM3604, TRAV17, 8430429K09RIK, VAULTRC5, FANCI, IGHV1-55, SLC25A13, SHCBP1, KCNMB4, NCAPG2, CDKN3, LDHB, VSIG10L, RTL8C, GM15452, 5330406M23RIK, HSPE1-PS2, IGKV1-135, KDM8, CENPE, GM45837, GM9887, IGLC3, TNFSF13B, TBC1D7, BIRC5, NARS2, GM10717, TRAV14-3, GM6644, TCRG-V3, MCOLN3, NEK6, GM36298, IGLV1, GM4924, GM3791, CDCA5, F730043M19RIK, RYK, TSGA10, NEIL3, 2010204K13RIK, ID3, MTERF2, HACL1, NRARP, AIRN, GM6565, CAMKMT, GM14117, 2810025M15RIK, RASGEF1B, TSPAN32, ADGRG1, PBK, FAM57A, GM36756, ZFP955A, SLC37A2, 4930532G15RIK, PLK4, IGKV4-59, CENPS, GM14305, ITM2A, GM14434, GM49172, GZMC, GM12808, SGO1, 3300002I08RIK, D630039A03RIK, CSTAD, GM39469, H3F3C, ABCG2, TIMP2, GM7805, CHN2, GM23419, IGLC1, TWSG1, AC183097.1, SUSD1, ARMC3, GM23245, ECT2, SNORA65, SPC24, SUPT3, TMEM147OS, ZFP119B, TRIP13, EXO1, MRM2, 1700001O22RIK, E130308A19RIK, GM20689, GM14494, POU2AF1, IFT22, GM16283, GM12184, TLR7, SLC22A15, GM15725, MIR8104, EPOP, CCNB1, GM49384, EOMES, TIGIT, HSPA4L, GNG11, CENPI, GM21738, ABHD1, LCLAT1, KIF18A, TBC1D4, APOBEC2, E2F8, PLS3, HAVCR2, DTL, PPFIBP1, TMLHE, KIF11, SNORD22, SLFN10-PS, TSPAN6, HGS, BUB1, GM43058, COX11, GM13597, ESCO2, 6720489N17RIK, TRAV6-5, SCRN2, CERS6, ZFP213, OSGIN1, AURKB, KIF2C, PVRIG, GM5931, ENPP5, TRAV7-5, ZFP599, RPL10-PS6, SLC25A29, RAD51, CRTAM, CCNB2, GM17173, GM26910, SERPINE2, MYB, GM33782, CKAP2, PBX4, GM4735, MAGEE1, GM26049, GM19196, LAG3, PDCD1LG2, GM15536, RAD51AP1, GM10226, SERP2, CCL3, TNIP3, GM11175, EEA1, NRN1, PFN2, GM11474, GM45236, MYBL2, IGHV6-3, BCL2A1C, KLRA7, SUSD2, TRAJ39, NEK2, CD200R1, MYPOP, GM17122, CENPF, CT010524.1, GAS2, GM10721, GM26527, D930028M14RIK, DYNLT1-PS1, PRSS2, NAT8F4, CD81, KLRA9, SRGAP3, GM14295, PDGFB, GM15706, GM10509, GM22579, GM37474, GM24539, CDC6, GM17764, MXD3, TRBV1, GM45778, GM26870, HMMR, EPHA3, AL731706.2, SPP1, MIR7058, AC164431.1, PXMP2, GM15443, GM10801, TMEM254C, GM45716, CELA1, 2310031A07RIK, JDP2, GM13509, BC035044, GM10719, DEPDC1A, 2810468N07RIK, GM10130, IGKV1-110, DUSP4, RPS11-PS1, GM10163, TMSB15L, SPO11, GCNT1, CPQ, GM16702, MT-TD, MIR8097, TROAP, GM49356, ATP1B1, 2010109A12RIK, GM9803, AKR1E1, NME4, PTGER2, MT-TR, TJP2, 6430590A07RIK, PPIC, FKBP10, ART2A-PS, PLXDC2, GM26860, MELK, ARL6, GM37376, UBASH3B, RAD54L, 1810062O18RIK, KNL1, TRPS1, FBXL15, ADRB1, LRIG1, GM11998, CCDC142OS, 5033430I15RIK, GM7666, GM24119, TCRG-C3, MT-TH, GM14048, TTK, TMEM273, BEND6, GM2800, TRAV15-1-DV6-1, BUB1B, MT-TK, EPHX1, GM14539, MIR22HG, GM7582, MIR6340, NINJ2, MAP6, SMS-PS, NUF2, HIST3H2BA, GM47586, TOX, SYNPO, POPDC2, CD244A, GM10108, 6330403K07RIK, CAR2, ZFAND4, TNFRSF9, GM10718, BCAT1, A630023P12RIK, GM37560, TCRG-V7, NAIP5, GM6851, PACSIN1, EPCAM, GM14125, GM35363, HIC1, D630008O14RIK, POC1A, MIR1931, CD83, ENPP2, GM32687, GM12854, GM10169, CXCR5, P2RX7, KCNIP3, GM16550, KLRA3, PHACTR2, SPRY1, GM20402, IGHD, GM10180, FCRL1, XCL1, GM47308, ITGAD, KNTC1, GM10736, 9630013D21RIK, NR4A2, GM44148, GM15433, MSL3L2, SNORA73B, MORN2, GM9790, GM15387, GM28942, GM17745, ICA1, SEPT4, MYO1E, 1700011L03RIK, MIR155HG, B4GALT4, GM24451, GM10182, IKZF2, DUSP14, CXXC5, EGR2, PDCD1, CYTH3, GM7334, AC122217.2, GM10157, CAAA01147332.1, IZUMO1R, GM26377, FAM69B, PRR5L, ART3, RASGEF1A, HIST1H1B, GM11518, CCL1, 4930417O13RIK, CD22, TNFRSF4, RGS16, GM10273, HIST1H2AP, GM10197, NEFH, CD200R2, GM11579, GPM6B, GLP1R, ST6GALNAC3, CD200, BACE2, A930002I21RIK, TG, IL1R2, 2900026A02RIK, PERP, IGHG3, GM37401, HMGN3, ITIH5, COCH, SOSTDC1, GM11454, GM6637, ZBTB32, BC002163, GM12338, OCIAD2, IL21, GM4956, 1700019D03RIK, TOX2, TNFSF4, NRGN, TRBV12-1, CCR6, PENK |
|  | Downregulation | SGK3, TREML4, BC005537, RPS6, GM17275, RPL9-PS4, GM11361, MT-ND2, CT010467.1, RPL13, GM37494, RPL37, RPS14, RPS11, GM9616, RPL18-PS2, RPS27RT, GM25890, ZC3H11A, TMEM154, RPS25, RASA4, BBS9, RPL7A, RPS27A-PS2, TET1, SELENOT, RTN4RL1, IL18BP, GM9843, TRBJ2-1, EMP1, RPL19-PS11, RPS15A-PS6, RPS18, MT-ND4, RPL26, RACK1, SLC17A9, RPS16-PS2, WDTC1, GM12191, SIPA1L3, ZFP652, RARG, APOE, LST1, MIR7115, ESM1, RPS9, RPL8, PPP1R3E, D17H6S53E, MT-ND5, RPL41, TNFRSF14, MIR1901, SLC25A23, GPNMB, GM22614, RPL32, ATXN1L, RPL10-PS3, GM48719, GPR183, CXCR6, 0610009E02RIK, SUN2, 1700088E04RIK, ACTB, RPL18A, TANC1, DPM3, FOSB, RPL24, ABHD15, MT-ND3, CD79B, EEF1B2, 4833403J16RIK, TNFRSF22, RPL19, IL18R1, DUSP7, GM19705, CLEC12A, TDRP, PBX2, BC051226, IGHV1-58, LYZ2, GM7299, KBTBD11, ADGRE4, FBRS, TRAV23, GM49553, ZSCAN26, GM48583, IFI208, GM35037, GM10241, PLAC8, GM3788, GM27640, ASS1, GPR34, GM7536, UBA52, HBB-BT, MIR142HG, RPL14, GPR132, RPL23, RPS13, CARD6, RPL36, PIM1, HBB-BS, HID1, BOLA2, RPL10A, SNHG12, GM5511, GM2423, RETNLG, GM3362, RPL22, GM10060, SERPINB1A, RPL14-PS1, GM9385, TRAJ17, GM4540, GM43434, RREB1, MT-TV, SEMA4B, FOSL2, SNORD42A, TRAJ32, MS4A6C, GRAP2, 4930486L24RIK, GM13166, CHD3, HP, TRAJ38, GGT1, SNORD35B, H2-AA, GM28935, H2-EB1, SIRPA, RPS20, H2-AB1, CD40LG, RPL23A, GM43302, C1QA, PMEPA1, GM28959, ITGB3, GM10522, GM25313, FHOD1, THY1, KCNJ8, C1QC, SNORD32A, RPL35A, MIR142B, PRSS12, PMEL, GM49521, RPLP1, GM32633, ZBTB4, ABHD2, RPL17-PS3, RPL13A, RPS10, TPT1, NUCB2, GM11808, RPL31, MT-CO1, RN7SK, GM47467, ATN1, GM22710, GM28872, BICRA, FOXO3, GM4149, LGMN, IFITM6, CFB, RPL37A, HCST, RPL30, RPL11, RPS23, RPS16, TLR6, OAS2, RPS7, GM17201, SH3BP5, PPP1R9B, CLEC4N, RPL36A, GM12013, GM37335, PIRB, H2-DMB1, MIR5121, SLC16A5, RARA, GM24452, GM22133, TRAJ6, STX1A, RPL23A-PS3, RPL9-PS6, 5830468F06RIK, RPS15, SKI, RPLP2, FOXO4, RPS12, BCL9L, GM10076, RPL34, CSF1R, GM2058, GM14303, RPS15A, GM26083, BASP1, TNFRSF26, SATB1, LCN4, UNC5A, G430095P16RIK, ZNRF3, RPS28, GM6472, RPL37RT, OPLAH, GM16069, GM10275, PLD4, GM28438, RPS15-PS2, RPS24, RPS5, FCGR3, AIF1, MIR7079, TXNIP, CD300E, GM6136, 1700047I17RIK2, S1PR1, HSD17B11, VAT1, TRAJ31, GM25939, AC113006.1, CXCR4, F2RL2, NEDD4L, ENG, SELENOP, RPS29, FAM78A, GM22077, MPEG1, RPL27A, GPC1, TRBJ1-3, RAPGEF4, GM22068, GM12929, GM25679, RPL38, GM26444, GM22634, SOCS2, CD300A, E030030I06RIK, GM29585, GM22317, SMAD3, SNORD14D, GM9830, HS3ST3B1, MIDN, HIST1H4N, TTYH3, RPS24-PS3, GM13625, ITGAM, SNORA64, G0S2, GM23804, IFITM3, GM24270, RNU1A1, GM29243, FAU, CDKN1B, RPL9-PS7, RNF144A, IFNGR1, GM12764, CCL6, PAG1, GM13461, TRAJ26, IFITM2, GM24245, MLANA, 7330423F06RIK, ITGAE, RPL27-PS3, PLAUR, TRAJ7, SPI1, TMEM176A, KLRB1C, MIR7067, ZBTB7B, IFNGR2, TMEM176B, TRAJ23, RNU3A, TNFRSF23, CTSH, C1QB, BBC3, GM20186, GPR141, GM44313, PLATR17, TRAJ42, CTDSP1, RPS15A-PS4, RBM3-PS, FCER1G, PILRA, ADGRE1, CD63, GM15491, SLC15A2, HEATR9, EAR2, RPS12-PS3, IGFBP4, GNG12, G530012D18RIK, GM23935, IGSF6, FAM241A, GM37691, RPL39, GM4208, MIR7072, RNU11, PLBD1, LAIR1, CLEC7A, NFAM1, GM10095, GM35035, GM25099, TFAP4, TGFBI, NUPR1, BACH1, GM14176, CLEC2D, CAR6, GM24407, ITGA1, RPL36-PS12, GM17041, NHSL2, GM27624, PF4, CHIL5, MS4A7, GM9320, AC169509.1, GM33104, ANXA1, MIR6989, GALNT10, SLCO3A1, RPL6L, RPS15A-PS5, GM7331, GM18852, IFI207, GZMM, GM10132, SLC6A19, TYROBP, RPS21, TRAJ45, FCGR1, TRGJ2, RPL17-PS9, GM15148, AA467197, MT-ND4L, SIRPB1C, CLEC4A1, RPL17-PS8, LY6I, RPL39-PS, GM10260, GM49342, DCT, APOBR, TRBV26, GM16534, TRAV13-2, MIR7686, GM9733, GM36161, RXRA, HSPA1B, GM28661, LY86, GGT5, GM45507, GM15429, SNHG9, IL7R, QPCT, 1700027A07RIK, TMA7-PS, GM43652, CLEC4A3, AC122413.1, GM2581, RNASE6, WFDC17, GM9844, TRAV7-6, GM10925, GM14414, RNU2-10, GM49346, TRBV20, GM14633, GM23971, TRDV4, GM24950, SPARCL1, GM23849, GM11966, CD300LD, I830127L07RIK, RPS10-PS2, IL15, GM24357, DKKL1, PTGDS, TRAV15N-2, TRAV15D-2-DV6D-2, GM37988, AQP3, MIR5125, GM10268, IL1RN, KLRA2, SNORD57, THRA, AXIN2, FCGRT, BLOC1S2-PS, LY6C2, ALOX5AP, CD300C2, TRIM30C, CD36, ADGRG5, IFI205, FCGR4, GM17134, GM25745, FES, TLE1, RPS13-PS1, IL1B, ZFP683, ST8SIA1, GM7618, LRRC75B, GM27486, GM18537, LYPD6B, CCL2, WFIKKN2, ITGAX, CCL8, SNORD58B, CLEC4A2, GM28437, TREML2, HSPA1A, GM47441, ARL4C, IFITM1, P2RY14, SNORD118, IL12RB2, TYRP1, GM27529, LY6G5B, GM27597, GM24265, LY6C1, GM27288, SLAMF8, GM13392, GM48909, GM27907, GM21887, SCARNA17, GM22513, GM27517, DDIT3, TRAJ47, FCGR2B, AC133103.5, EYA2, GM20529, GM48990, GM46209, GM10193, PILRB2, CCR2, CNR2, VIPR1, GM11702, TRAV14N-3, SNORD104, SNORD13, TRAJ9, GM44419, TRAJ58, TRAJ49, GM26825, MIR3064, CYP2S1, GM10222, PILRB1, GM15459, GM14039, RNU3B2, GM10263, RNU3B4, RNU3B3, RNU3B1, RPL17-PS10, AY036118, TRAJ37, AQP9, MT-TS1, TRDJ1, TRAJ48 |
| GO_upregulation | Response to stimulus | Abcg2, Adrb1, Alcam, Apitd1, Arhgap39, Arl6, Atp1b1, Aurkb, Car2, Ccl1, Ccl3, Ccna2, Ccnb1, Ccr6, Cd160, Cd200r1, Cd200r2, Cd22, Cd38, Cd81, Cd83, Cdca5, Cdkn3, Cela1, Chn2, Clspn, Coch, Cpq, Crtam, Csf1, Cxcr5, Depdc1a, Dtl, Ect2, Egr2, Eid2, Enpp2, Eomes, Epcam, Epha3, Ephx1, Esco2, Exo1, Fanci, Fcna, Fcrl1, Fen1, Folr4, Gcnt1, Gem, Glp1r, Gng11, Gpr56, Gzmc, Havcr2, Hells, Hic1, Hspa4l, Id3, Ifi44, Ifng, Il1r2, Il21, Irf8, Itgad, Itm2a, Kcnip3, Kcnmb4, Lag3, Litaf, Melk, Mrnip, Myb, Mybl2, Myo1e, Naip5, Nefh, Neil3, Nek6, Ninj2, Nr4a2, Nrarp, Nrbf2, Nrgn, Nrip1, P2rx7, Pacsin1, Pbk, Pdgfb, Penk, Perp, Phb, Plscr1, Prnp, Prr5l, Prss2, Ptger2, Rad51, Rad51ap1, Rad54l, Rasgef1b, Rgs16, Rnf43, Rph3al, Ryk, Serp2, Serpine2, Shcbp1, Sirt5, Slc25a13, Slc25a29, Sostdc1, Spo11, Spp1, Srgap3, Stk3, Stx11, Susd2, Synpo, Tbc1d4, Tbc1d7, Tesk2, Tg, Timp2, Tjp2, Tlr7, Tnfrsf4, Tnfrsf9, Tnfsf13b, Tnfsf4, Top2a, Traip, Trip13, Trps1, Tspan32, Tspan6, Twsg1, Ubash3b, Uhrf1, Xcl1 |
|  | Signal transduction | Adrb1, Arhgap39, Arl6, Atp1b1, Car2, Ccl1, Ccl3, Ccna2, Ccr6, Cd160, Cd200r1, Cd200r2, Cd22, Cd38, Cd81, Cd83, Cela1, Chn2, Csf1, Cxcr5, Depdc1a, Dtl, Ect2, Eid2, Epcam, Epha3, Fcna, Fcrl1, Folr4, Gem, Glp1r, Gng11, Gpr56, Gzmc, Havcr2, Hic1, Ifng, Il1r2, Il21, Itgad, Lag3, Melk, Myo1e, Nek6, Nr4a2, Nrarp, Nrgn, P2rx7, Pacsin1, Pdgfb, Penk, Perp, Phb, Plscr1, Prnp, Prr5l, Ptger2, Rasgef1b, Rgs16, Rnf43, Ryk, Serp2, Shcbp1, Sostdc1, Srgap3, Stk3, Tesk2, Tjp2, Tlr7, Tnfrsf4, Tnfrsf9, Traip, Trps1, Tspan32, Tspan6, Twsg1, Ubash3b, Xcl1 |
|  | Cell cycle | Apitd1, Aurkb, Birc5, Bub1, Bub1b, Casc5, Ccna2, Ccnb1, Ccnb2, Ccne2, Ccnf, Cdc6, Cdca5, Cdca8, Cdkn3, Cenpe, Cep55, Ckap2, Clspn, E2f8, Ect2, Esco2, Exo1, Fanci, Fbxl15, Gas2, Gem, Gm12184, Hells, Ifng, Kdm8, Kif11, Kif18a, Kif18b, Kif2c, Kntc1, Melk, Mis18bp1, Mrnip, Myb, Mybl2, Ncapg2, Nek2, Nek6, Nuf2, Pard6a, Pbk, Plk4, Poc1a, Rad51, Sept4, Sgol1, Ska3, Spc24, Spo11, Spry1, Top2a, Trip13, Ttk, Uhrf1 |
|  | Immune system process | Alcam, Ccl1, Ccl3, Ccnb2, Ccr6, Cd38, Cd83, Crtam, Csf1, Cxcr5, Enpp2, Eomes, Esco2, Exo1, Fcna, Fcrl1, Gcnt1, Gm20388, Havcr2, Hells, Ifng, Il21, Irf8, Itgad, Itm2a, Melk, Ms4a1, Myb, Myo1e, Naip5, Ncapg2, P2rx7, Pdcd1, Pdgfb, Pgm3, Plscr1, Spp1, Stk3, Stx11, Susd2, Tlr7, Tnfrsf4, Tnfrsf9, Tnfsf13b, Tnfsf4, Top2a, Tox, Twsg1, Xcl1, Zbtb32 |
|  | Cell division | Apitd1, Aurkb, Birc5, Bub1, Bub1b, Casc5, Ccna2, Ccnb1, Ccnb2, Ccne2, Ccnf, Cdc6, Cdca5, Cdca8, Cenpe, Cep55, Ckap2, Ect2, Hells, Kif11, Kif18b, Kif2c, Kntc1, Mis18bp1, Myb, Ncapg2, Nek2, Nek6, Nuf2, Pard6a, Sept4, Sgol1, Ska3, Spc24, Top2a |
|  | Mitotic cell cycle process | Aurkb, Birc5, Bub1, Bub1b, Ccna2, Ccnb1, Ccne2, Cdca5, Cdca8, Cdkn3, Cenpe, Cep55, Ckap2, Clspn, Ect2, Fanci, Fbxl15, Kdm8, Kif11, Kif18a, Kif18b, Kif2c, Kntc1, Mrnip, Myb, Mybl2, Nek2, Poc1a, Rad51, Sgol1, Spry1, Top2a, Trip13, Ttk |
|  | Regulation of cytokine production | Ccl3, Cd226, Cd83, Crtam, Fcna, Gpatch3, Havcr2, Ifng, Il1r2, Il21, Irf8, Lag3, Litaf, Naip5, P2rx7, Pdcd1lg2, Prnp, Tigit, Tlr7, Tnfrsf4, Tnfrsf9, Tnfsf4, Traip, Tspan6, Twsg1, Xcl1, Zbtb32 |
|  | Regulation of cell activation | Cd200, Cd226, Cd38, Cd81, Cd83, Havcr2, Ifng, Il21, Lag3, Myb, Nrarp, Pdcd1lg2, Pdgfb, Plscr1, Prnp, Serpine2, Tigit, Tnfrsf4, Tnfsf13b, Tnfsf4, Tox, Tspan32, Twsg1, Ubash3b, Xcl1 |
|  | Defense response | Ccl3, Cd226, Coch, Crtam, Fcna, Gpatch3, Havcr2, Ifng, Il1r2, Il21, Lag3, Pbk, Penk, Phb, Plscr1, Tlr7, Tnfsf4, Tspan32, Tspan6, Xcl1 |
|  | Cellular response to lipid | Ccl3, Ccna2, Ccnb1, Epha3, Ephx1, Havcr2, Id3, Ifng, Irf8, Litaf, Nr4a2, Nrip1, Penk, Phb, Plscr1, Ptger2, Spp1, Tnfsf4 |
|  | Regulation of chromosome organization | Aurkb, Birc5, Bub1, Bub1b, Ccnb1, Cdc6, Cdca5, Cenpe, Fen1, H3f3a, Jdp2, Myb, Nek2, Nek6, Top2a, Trip13, Ttk, Uhrf1 |
|  | DNA repair | Apitd1, Cdca5, Clspn, Dtl, Esco2, Exo1, Fanci, Fen1, Mrnip, Neil3, Rad51, Rad51ap1, Rad54l, Spo11, Trip13, Uhrf1 |
|  | Regulation of leukocyte differentiation | Car2, Ccl3, Cd83, Csf1, Ifng, Il21, Myb, Nrarp, Tjp2, Tnfsf4, Tox, Ubash3b |
|  | Regulation of T cell proliferation | Havcr2, Ifng, Il21, Pdcd1lg2, Prnp, Tnfsf13b, Tnfsf4, Twsg1, Xcl1 |
|  | Leukocyte chemotaxis | Ccl1, Ccl3, Cxcr5, Ifng, Pdgfb, Spp1, Xcl1 |
| GO_downregulation | Cellular process | AA467197, Abhd15, Abhd2, Actb, Aif1, Alox5ap, Anxa1, Apobr, Apoe, Aqp3, Ass1, Atn1, Atxn1l, Axin2, Bach1, Basp1, Bbc3, Bbs9, Bcl9l, Bola2, Car6, Ccl2, Ccl6, Ccl8, Ccr2, Cd300a, Cd36, Cd40lg, Cd63, Cd79b, Cdkn1b, Clec2d, Clec4a2, Clec7a, Cnr2, Csf1r, Ctdsp1, Ctsh, Cxcr4, Cxcr6, Cyp2s1, Dct, Ddit3, Dpm3, Dusp7, ENSMUSG00000050345, Ear2, Eef1b2, Emp1, Emr1, Emr4, Eng, Eya2, F2rl2, Fau, Fcer1g, Fcgr1, Fcgr2b, Fcgr3, Fcgr4, Fcgrt, Fes, Fosb, Fosl2, Foxo3, Foxo4, G0s2, Galnt10, Ggt1, Ggt5, Gm9830, Gnb2l1, Gng12, Gpc1, Gpnmb, Gpr114, Gpr132, Gpr141, Gpr183, Gpr34, Grap2, H2-Ab1, H2-DMb1, Hbb-bt, Hcst, Heatr9, Hp, Hs3st3b1, Hsd17b11, Hspa1a, Hspa1b, Ifi205, Ifitm3, Ifngr1, Il12rb2, Il15, Il18bp, Il18r1, Il1b, Il1rn, Il7r, Itga1, Itgae, Itgam, Itgax, Itgb3, Kbtbd11, Kcnj8, Klrb1c, Lgmn, Lst1, Lyz2, Nedd4l, Nfam1, Nhsl2, Nucb2, Nupr1, Oas2, Oplah, P2ry14, Pag1, Pbx2, Pf4, Pilra, Pilrb1, Pilrb2, Pim1, Pirb, Plac8, Plaur, Pld4, Pmel, Ppp1r3e, Ppp1r9b, Prss12, Ptgds, Qpct, Rapgef4, Rara, Rarg, Rasa4, Retnlg, Rnase6, Rnf144a, Rpl10-ps3, Rpl10a, Rpl11, Rpl13, Rpl13a, Rpl14, Rpl17, Rpl18a, Rpl19, Rpl22, Rpl23, Rpl23a, Rpl24, Rpl26, Rpl27-ps3, Rpl27a, Rpl30, Rpl31, Rpl32, Rpl34, Rpl35a, Rpl36, Rpl36a, Rpl37, Rpl37a, Rpl38, Rpl39, Rpl41, Rpl7a, Rpl8, Rplp1, Rplp2, Rps10, Rps11, Rps12-ps3, Rps13, Rps14, Rps15, Rps15a, Rps16, Rps18, Rps20, Rps21, Rps23, Rps24, Rps25, Rps28, Rps29, Rps5, Rps6, Rps7, Rps9, Rreb1, Rtn4rl1, Rxra, S1pr1, Satb1, Selt, Sema4b, Sepp1, Sgk3, Sh3bp5, Sipa1l3, Sirpa, Ski, Slamf8, Slc17a9, Slc25a23, Smad3, Snrpa, Socs2, Sparcl1, Spi1, St8sia1, Stx1a, Sun2, Tanc1, Tet1, Tgfbi, Thra, Thy1, Tle1, Tlr6, Tmem176b, Tnfrsf22, Tnfrsf23, Tnfrsf26, Tpt1, Treml2, Treml4, Txnip, Tyrobp, Tyrp1, Uba52, Unc5a, Vipr1, Wdtc1, Wfikkn2, Ywhaq, Zbed6, Zbtb4, Zbtb7b, Zfp652, Zfp683, Znrf3, Zscan26, mt-Atp6, mt-Co1, mt-Nd2, mt-Nd3, mt-Nd4, mt-Nd4l, mt-Nd5 |
|  | Metabolic process | AA467197, Abhd15, Abhd2, Alox5ap, Anxa1, Apobr, Apoe, Ass1, Atn1, Atxn1l, Axin2, Bach1, Bcl9l, Bola2, C1qa, C1qb, C1qc, Car6, Cd36, Cd40lg, Cdkn1b, Cfb, Csf1r, Ctdsp1, Ctsh, Cyp2s1, Dct, Ddit3, Dpm3, Dusp7, ENSMUSG00000050345, Ear2, Eef1b2, Eng, Eya2, Fau, Fcer1g, Fes, Fosb, Fosl2, Foxo3, Foxo4, Galnt10, Ggt1, Ggt5, Gm9830, Gnb2l1, Gpc1, Grap2, Hcst, Hs3st3b1, Hsd17b11, Hspa1a, Ifi205, Il12rb2, Il15, Il1b, Il1rn, Kbtbd11, Lgmn, Lyz2, Nedd4l, Nucb2, Nupr1, Oas2, Oplah, Pbx2, Pim1, Plbd1, Pld4, Pmel, Ppp1r3e, Ppp1r9b, Prss12, Ptgds, Qpct, Rara, Rarg, Rnase6, Rnf144a, Rpl10-ps3, Rpl10a, Rpl11, Rpl13, Rpl13a, Rpl14, Rpl17, Rpl18a, Rpl19, Rpl22, Rpl23, Rpl23a, Rpl24, Rpl26, Rpl27-ps3, Rpl27a, Rpl30, Rpl31, Rpl32, Rpl34, Rpl35a, Rpl36, Rpl36a, Rpl37, Rpl37a, Rpl38, Rpl39, Rpl41, Rpl7a, Rpl8, Rplp1, Rplp2, Rps11, Rps12-ps3, Rps13, Rps14, Rps15, Rps15a, Rps16, Rps18, Rps20, Rps21, Rps23, Rps24, Rps28, Rps29, Rps5, Rps6, Rps7, Rps9, Rreb1, Rxra, Satb1, Selt, Sepp1, Sgk3, Ski, Smad3, Snrpa, Socs2, Spi1, St8sia1, Tet1, Thra, Thy1, Tle1, Tlr6, Txnip, Tyrp1, Uba52, Vat1, Wdtc1, Zbed6, Zbtb4, Zbtb7b, Zfp652, Zfp683, Znrf3, Zscan26, mt-Atp6, mt-Co1, mt-Nd2, mt-Nd3, mt-Nd4, mt-Nd4l, mt-Nd5 |
|  | Signaling | Abhd2, Aif1, Anxa1, Apoe, Axin2, Bbc3, Bcl9l, Ccl2, Ccl6, Ccl8, Ccr2, Cd300a, Cd36, Cd40lg, Cd79b, Cdkn1b, Clec2d, Cnr2, Csf1r, Ctsh, Cxcr4, Cxcr6, Ddit3, Emr1, Emr4, Eng, Eya2, F2rl2, Fcer1g, Fcgr1, Fcgr2b, Fcgr3, Fcgr4, Fcgrt, Fes, Foxo3, Foxo4, G0s2, Gnb2l1, Gng12, Gpnmb, Gpr114, Gpr132, Gpr141, Gpr183, Gpr34, Grap2, Hp, Ifitm3, Ifngr1, Il12rb2, Il15, Il18r1, Il1b, Il1rn, Il7r, Itga1, Itgae, Itgam, Itgax, Itgb3, Klrb1c, Nfam1, Nupr1, Oas2, P2ry14, Pag1, Pf4, Pilra, Pilrb1, Pilrb2, Pim1, Pirb, Plaur, Ppp1r9b, Rapgef4, Rara, Rarg, Rasa4, Rpl26, Rps6, Rreb1, Rtn4rl1, Rxra, S1pr1, Selt, Sgk3, Sh3bp5, Ski, Slamf8, Smad3, Socs2, Sparcl1, Stx1a, Thra, Thy1, Tle1, Tlr6, Tnfrsf22, Tnfrsf23, Tnfrsf26, Treml2, Txnip, Tyrobp, Unc5a, Vipr1, Wfikkn2, Ywhaq, Znrf3 |
|  | System development | Actb, Aif1, Anxa1, Apoe, Aqp3, Ass1, Atn1, Atxn1l, Axin2, Basp1, Bcl9l, C1qb, Ccl2, Ccr2, Cd40lg, Cd79b, Cdkn1b, Csf1r, Ctdsp1, Ctsh, Cxcr4, Dct, Ddit3, Eng, Esm1, Fcer1g, Fcgr2b, Fes, Fosl2, Foxo3, Foxo4, Gng12, Gpc1, Gpnmb, Gpr183, H2-Ab1, Hbb-bt, Heatr9, Hp, Ifitm2, Il15, Il18r1, Il1b, Il7r, Itga1, Itgam, Kcnj8, Lst1, Nedd4l, Nfam1, Nupr1, Pirb, Plaur, Pld4, Ppp1r9b, Rapgef4, Rara, Rarg, Rpl10-ps3, Rpl13a, Rpl19, Rpl22, Rpl24, Rpl32, Rpl38, Rps14, Rps6, Rps7, Rtn4rl1, Rxra, S1pr1, Satb1, Selt, Sema4b, Sepp1, Sipa1l3, Sirpa, Ski, Smad3, Socs2, Spi1, Sun2, Tgfbi, Thra, Thy1, Tnfrsf22, Tnfrsf23, Tnfrsf26, Txnip, Tyrobp, Unc5a, Wfikkn2, Zbtb7b, Zfp683, mt-Co1, mt-Nd4 |
|  | Macromolecule biosynthetic process | Apoe, Atn1, Atxn1l, Bach1, Bcl9l, Ddit3, Dpm3, Eef1b2, Eya2, Fau, Fosb, Fosl2, Foxo3, Foxo4, Galnt10, Ggt1, Ggt5, Gnb2l1, Ifi205, Il1b, Nupr1, Pbx2, Rara, Rarg, Rpl10-ps3, Rpl10a, Rpl11, Rpl13, Rpl13a, Rpl14, Rpl17, Rpl18a, Rpl19, Rpl22, Rpl23, Rpl23a, Rpl24, Rpl26, Rpl27-ps3, Rpl27a, Rpl30, Rpl31, Rpl32, Rpl34, Rpl35a, Rpl36, Rpl36a, Rpl37, Rpl37a, Rpl38, Rpl39, Rpl41, Rpl7a, Rpl8, Rplp1, Rplp2, Rps11, Rps12-ps3, Rps13, Rps14, Rps15, Rps15a, Rps16, Rps18, Rps20, Rps21, Rps23, Rps24, Rps28, Rps29, Rps5, Rps6, Rps7, Rps9, Rreb1, Rxra, Satb1, Ski, Smad3, Spi1, St8sia1, Tet1, Thra, Tle1, Txnip, Uba52, Zbed6, Zbtb4, Zbtb7b, Zfp652, Zfp683, Zscan26 |
|  | Positive regulation of response to stimulus | Aif1, Alox5ap, Anxa1, Bbc3, C1qa, C1qb, C1qc, Ccl2, Ccl6, Ccl8, Ccr2, Cd36, Cd40lg, Cd63, Cd79b, Cfb, Clec4n, Csf1r, Ctsh, Cxcr4, Ddit3, Eng, Esm1, Eya2, F2rl2, Fcer1g, Fcgr1, Fcgr2b, Fcgr3, Foxo3, G0s2, Gnb2l1, Gpnmb, Gpr183, H2-Ab1, Hcst, Igfbp4, Il15, Il18r1, Il1b, Il1rn, Il7r, Itga1, Itgam, Itgb3, Klrb1c, Lgmn, Ly86, Nfam1, Pag1, Pf4, Plaur, Rara, Rpl11, Rpl23, Rpl26, Rps7, Rreb1, S1pr1, Smad3, Socs2, Stx1a, Thra, Thy1, Tlr6, Treml4, Zbtb7b |
|  | Regulation of molecular function | Actb, Alox5ap, Anxa1, Apoe, Axin2, Bbc3, Ccl2, Ccl6, Ccl8, Cd300a, Cd36, Cd40lg, Cdkn1b, Cnr2, Csf1r, Ctsh, Ddit3, Dusp7, Eng, Fbrs, Fcgr2b, Gnb2l1, Gpnmb, Hp, Hspa1b, Il15, Il18bp, Il18r1, Il1b, Il1rn, Itga1, Itgam, Itgb3, Lgmn, Lypd6b, Midn, Nedd4l, Nfam1, Pf4, Pim1, Plaur, Ppp1r9b, Rapgef4, Rara, Rasa4, Retnlg, Rpl11, Rpl23, Rplp1, Rps7, Rtn4rl1, S1pr1, Serpinb1a, Sgk3, Sh3bp5, Sipa1l3, Ski, Slamf8, Smad3, Socs2, Stx1a, Thra, Thy1, Tlr6, Txnip, Wdtc1, Wfikkn2 |
|  | Translation | Eef1b2, Fau, Ggt1, Ggt5, Gnb2l1, Rpl10-ps3, Rpl10a, Rpl11, Rpl13, Rpl13a, Rpl14, Rpl17, Rpl18a, Rpl19, Rpl22, Rpl23, Rpl23a, Rpl24, Rpl26, Rpl27-ps3, Rpl27a, Rpl30, Rpl31, Rpl32, Rpl34, Rpl35a, Rpl36, Rpl36a, Rpl37, Rpl37a, Rpl38, Rpl39, Rpl41, Rpl7a, Rpl8, Rplp1, Rplp2, Rps11, Rps12-ps3, Rps13, Rps14, Rps15, Rps15a, Rps16, Rps18, Rps20, Rps21, Rps23, Rps24, Rps28, Rps29, Rps5, Rps6, Rps7, Rps9, Uba52 |
|  | Regulation of cell death | Aif1, Anxa1, Apoe, Axin2, Bbc3, Cd36, Cd40lg, Cdkn1b, Csf1r, Ctsh, Cxcr4, Ddit3, Dkkl1, Eya2, Fcer1g, Fcgr2b, Foxo3, G0s2, Gnb2l1, Gpnmb, Hp, Hspa1b, Il1b, Il1rn, Il7r, Itga1, Itgam, Itgb3, Lgmn, Nupr1, Pf4, Pim1, Plac8, Plaur, Rara, Rarg, Rpl10-ps3, Rpl11, Rpl26, Rps29, Rps6, Rps7, Rxra, Sgk3, Smad3, Thra, Tle1, Tlr6, Tnfrsf22, Tnfrsf23, Tnfrsf26, Tpt1, Txnip |
|  | Regulation of phosphorylation | Actb, Aif1, Apoe, Axin2, Ccl2, Ccl6, Ccl8, Cd300a, Cd36, Cd40lg, Cdkn1b, Csf1r, Ctdsp1, Cxcr4, Dusp7, Eng, Fcgr2b, Gnb2l1, Gpnmb, Gpr183, Hbb-bt, Igfbp4, Il15, Il1b, Il1rn, Itga1, Itgb3, Midn, Pim1, Plaur, Pmepa1, Ppp1r9b, Rara, Rplp1, Rtn4rl1, Sh3bp5, Slamf8, Slc25a23, Socs2, Thy1, Tlr6 |
|  | Positive regulation of immune system process | Aif1, Anxa1, Aqp3, Atxn1l, C1qa, C1qb, C1qc, Ccl2, Ccr2, Cd300a, Cd36, Cd40lg, Cd79b, Cfb, Csf1r, Fcer1g, Fcgr1, Fcgr2b, Fcgr3, Fes, Foxo3, Gpr183, H2-Aa, H2-Ab1, Il15, Il18r1, Il1b, Il7r, Itgam, Itgb3, Klrb1c, Lgmn, Nfam1, Pf4, Rara, Thy1, Tlr6, Treml4, Zbtb7b |
|  | Locomotion | Actb, Anxa1, Atn1, Ccl2, Ccl6, Ccl8, Ccr2, Cd63, Cnr2, Csf1r, Cxcr4, Cxcr6, Ear2, Eng, Fcer1g, Fcgr3, Fes, Gpr183, Grap2, Il1b, Itga1, Itgam, Itgb3, Pf4, Ppp1r9b, Retnlg, Rpl13a, Rpl24, S1pr1, Sema4b, Sirpa, Slamf8, Sun2, Tnfrsf22, Tnfrsf23, Tnfrsf26, Unc5a |
|  | Cell motility | Actb, Anxa1, Atn1, Ccl2, Ccl6, Ccl8, Ccr2, Cd63, Cnr2, Cxcr4, Eng, Fcer1g, Fcgr3, Fes, Gpr183, Grap2, Il1b, Itga1, Itgam, Itgb3, Pf4, Ppp1r9b, Retnlg, Rpl13a, S1pr1, Sirpa, Slamf8, Sun2 |
|  | Cell migration | Anxa1, Atn1, Ccl2, Ccl6, Ccl8, Ccr2, Cd63, Cnr2, Cxcr4, Eng, Fcer1g, Fcgr3, Fes, Gpr183, Grap2, Il1b, Itga1, Itgam, Itgb3, Pf4, Ppp1r9b, Retnlg, Rpl13a, S1pr1, Sirpa, Slamf8, Sun2 |
|  | Regulation of vesicle-mediated transport | Anxa1, Apoe, Ccl2, Ccr2, Cd300a, Cd36, Cd63, Fcer1g, Fcgr1, Fcgr2b, Fcgr3, Fes, Gnb2l1, Il1b, Itgam, Nedd4l, Rapgef4, Sirpa, Stx1a |
