# Supplementary material for: Transcriptome analysis of Homo sapiens and Mus musculus reveals mechanisms of CD8+ T cell exhaustion caused by different factors
Source: PLoS One. 2022 Sep 9;17(9):e0274494. doi: 10.1371/journal.pone.0274494 (PMC9462770; doi:10.1371/journal.pone.0274494)
Supplement: S3 Table — (DOCX) [file pone.0274494.s009.docx]

**S3 Table. Gene list of the red module in Fig 3.**

|  | **Gene** |
| --- | --- |
| Red module | MAD2L1, CDKN2C, FAM111B, EZH2, CCNB1, KIAA1324, CDCA7, CAMK1, CENPU, KNL1, CHN1, ATAD5, CKS1B, TP53INP1, PTTG1, KCNK5, CYB5B, POLA2, CHAF1B, DHFR, IFI16, CD70, MAGEH1, FANCG, CTSO, NAB1, CDC45, HMGB2, ACP5, MAST4, TBC1D4, TCF19, TNFSF10, SLC43A3, POLE2, ADRB2, MXI1, CCNB2, ATP8B4, CENPN, GPD2, RHOC, CDCA3, TMEM107, TRAF5, C3orf14, KIF20B, TIMMDC1, TROAP, AC090152.1, PARPBP, UAP1L1, ARHGAP11A, GLDC, CCNA2, SMC4, ACOT7, AGFG1, TMPO, RFC2, FAM3C2, CD200R1, PAM, TMEM155, SNAP47, BUB1B, KIF23, DONSON, CDC20, GALNT1, NUF2, CENPK, HMGN2P5, WDR34, PLK4, CXCL13, C12orf57, BIRC5, CENPH, FBXO5, LMCD1, SHCBP1, NCAPG, BRCA1, ATP10D, THSD8, GZMM, INPP5F, TOP2A, MIR4435-2HG, DTYMK, SLF1, SLC1A4, RNASEH2A, CIP2A, ZBED2, MCM5, HAVCR2, DNAJC9, VDR, CENPE, CHST12, SGO2, ETV1, SGO1, GCNT1, UBE2C, CCNE2, RAD51, CLSPN, CKS2, SLC25A46, MCM2, GINS2, NDC80, SRGAP3, DTL, CCDC28A, TMEM106C, PLK1, CD82, TPX2, KIF22, CDCA5, NFIL3, SRGAP2, TYMS, PRC1, NUSAP1, DGKH, KRT86, SPAG5, AURKA, CORO1C, DUSP4, DSN1, PRDM1, ARL3, PMAIP1, ZWINT, AC241952.1, CKAP2, TIGIT, CDK2, SYT11, YWHAH, CDC6, MTMR8, VCAM1, CBR3, AGMAT, CENPM, TIMELESS, TRIB1, CXCR6, STMN1, ASF1B, PPIAP45, CDK1, ARL2BP, HIRIP3, CENPW, FANCI, TOX, CD2BP2, INPP1, AC087632.1, SSH1, SNRNP25, WDHD1, MSH2, MCM7, ORC6, FAM3C, PSAT1, TSC22D1, MKI67, GPR137B, CDKN3, CPNE2, ARHGAP11B, PCNA, FABP5, CERS6, KIF2C, FKBP1A, ZNF79, AFAP1L2, MYEF2, CSGALNACT1, DUT, BTLA, C5orf30, UHRF1, BHLHE40-AS1, PMVK, HMGB1P5, CCDC141, PKMYT1, CLECL1, SMC2, PHTF2, ICOS, SKA2, LSP1, AURKB, PHGDH, E2F1, ECT2, METTL8, LIMS1, IFI27L1, GMNN, RDH10, CENPF, CD27, GEM, RACGAP1, MYBL2, CENPQ, RFC4, GAPDH, PDCD1, TK1, ACSL1, UBE2T, PCLAF, AC243829.4, HMGN2, CHEK1, PPIF, GALM, SARDH, SNX9, RAD51AP1, MYO7A, HIST1H2AH, TTC24, FABP5P7, RRM2, MELK, GGH, UBR7, NCAPD2, NCAPG2, CDCA8, ADAM28, TPI1, TUBA1B, SCCPDH, RAB38, AC006064.4, MCM6, PLPP1, FUT8, TRIP6, BUB1, CTLA4, TNFSF4, ENTPD1, FEN1, CDT1, MCM3 |
