# Supplementary material for: Transcriptome analysis of Homo sapiens and Mus musculus reveals mechanisms of CD8+ T cell exhaustion caused by different factors
Source: PLoS One. 2022 Sep 9;17(9):e0274494. doi: 10.1371/journal.pone.0274494 (PMC9462770; doi:10.1371/journal.pone.0274494)
Supplement: S4 Table — (DOCX) [file pone.0274494.s010.docx]

**S4 Table. Enriched gene sets of *Homo sapiens* and *Mus musculus*.**

| **Species** | **Gene sets** | **ES** | **NES** | **NOM p-val** |
| --- | --- | --- | --- | --- |
| *Homo sapiens* | GSE9650_EXHAUSTED_VS_MEMORY_CD8_TCELL_UP | -0.49170125 | -1.8977695 | 0 |
|  | GSE9650_NAIVE_VS_EFF_CD8_TCELL_DN | -0.47732887 | -1.8457063 | 0 |
|  | GSE41867_DAY6_VS_DAY8_LCMV_CLONE13_EFFECTOR_CD8_TCELL_UP | -0.49215046 | -1.9136045 | 0 |
|  | GSE24081_CONTROLLER_VS_PROGRESSOR_HIV_SPECIFIC_CD8_TCELL_DN | -0.53261566 | -1.8878177 | 0.002053388 |
|  | GSE41867_NAIVE_VS_DAY6_LCMV_ARMSTRONG_EFFECTOR_CD8_TCELL_DN | -0.3889367 | -1.5352044 | 0.004219409 |
|  | GSE9650_EFFECTOR_VS_MEMORY_CD8_TCELL_UP | -0.44736725 | -1.7990183 | 0.00814664 |
|  | GSE9650_NAIVE_VS_EXHAUSTED_CD8_TCELL_DN | -0.40583837 | -1.6110837 | 0.008403362 |
|  | GSE9650_EFFECTOR_VS_EXHAUSTED_CD8_TCELL_DN | -0.42118782 | -1.6291802 | 0.008583691 |
|  | GSE26495_NAIVE_VS_PD1HIGH_CD8_TCELL_DN | -0.4795171 | -1.7549728 | 0.008658009 |
|  | GSE41867_DAY6_EFFECTOR_VS_DAY30_EXHAUSTED_CD8_TCELL_LCMV_CLONE13_UP | -0.40014634 | -1.5713226 | 0.014522822 |
|  | GSE9650_GP33_VS_GP276_LCMV_SPECIFIC_EXHAUSTED_CD8_TCELL_UP | -0.37313476 | -1.4532682 | 0.042372882 |
|  | GSE26495_NAIVE_VS_PD1LOW_CD8_TCELL_DN | -0.41625872 | -1.564878 | 0.049676027 |
| *Mus musculus* | GSE41867_NAIVE_VS_EFFECTOR_CD8_TCELL_UP | -0.5094074 | -1.7294925 | 0 |
|  | GSE41867_NAIVE_VS_DAY8_LCMV_EFFECTOR_CD8_TCELL_UP | -0.48712343 | -1.6814346 | 0 |
|  | GSE9650_EFFECTOR_VS_EXHAUSTED_CD8_TCELL_DN | -0.47111788 | -1.60668 | 0 |
|  | GSE9650_NAIVE_VS_MEMORY_CD8_TCELL_UP | -0.44798443 | -1.587172 | 0.003960396 |
|  | GSE41867_DAY8_EFFECTOR_VS_DAY30_EXHAUSTED_CD8_TCELL_LCMV_CLONE13_UP | -0.4629008 | -1.7290453 | 0.004 |
|  | GSE9650_EXHAUSTED_VS_MEMORY_CD8_TCELL_UP | -0.54677474 | -1.7747765 | 0.01010101 |
|  | GSE41867_NAIVE_VS_DAY15_LCMV_EFFECTOR_CD8_TCELL_UP | -0.4683547 | -1.6239688 | 0.011952192 |
|  | GSE9650_EFFECTOR_VS_MEMORY_CD8_TCELL_UP | -0.52175516 | -1.8219271 | 0.012024048 |
|  | GSE41867_NAIVE_VS_DAY15_LCMV_CONE13_EFFECTOR_CD8_TCELL_DN | -0.4551377 | -1.60099 | 0.017578125 |
|  | GSE41867_DAY6_VS_DAY8_LCMV_ARMSTRONG_EFFECTOR_CD8_TCELL_UP | -0.38916937 | -1.492486 | 0.01984127 |
|  | GSE41867_NAIVE_VS_EFFECTOR_CD8_TCELL_DN | -0.49762318 | -1.684801 | 0.023622047 |
|  | KAMMINGA_EZH2_TARGETS | -0.8049297 | -1.5981472 | 0.026804123 |
|  | GSE41867_NAIVE_VS_DAY30_LCMV_ARMSTRONG_MEMORY_CD8_TCELL_UP | -0.4489148 | -1.5095041 | 0.030241935 |
|  | GSE41867_DAY8_EFFECTOR_VS_DAY30_MEMORY_CD8_TCELL_LCMV_ARMSTRONG_UP | -0.45777133 | -1.5747974 | 0.031809144 |
|  | GSE41867_NAIVE_VS_DAY15_LCMV_ARMSTRONG_EFFECTOR_CD8_TCELL_UP | -0.3866527 | -1.4964194 | 0.032719836 |
|  | GSE24026_PD1_LIGATION_VS_CTRL_IN_ACT_TCELL_LINE_DN | -0.49915084 | -1.6296711 | 0.03629032 |
|  | GSE9650_NAIVE_VS_EXHAUSTED_CD8_TCELL_DN | -0.39481124 | -1.4843673 | 0.045548655 |
