# Supplementary material for: Transcriptome analysis of Homo sapiens and Mus musculus reveals mechanisms of CD8+ T cell exhaustion caused by different factors
Source: PLoS One. 2022 Sep 9;17(9):e0274494. doi: 10.1371/journal.pone.0274494 (PMC9462770; doi:10.1371/journal.pone.0274494)
Supplement: S5 Table — (DOCX) [file pone.0274494.s011.docx]

**S5 Table. Overlap genes between the four enriched gene sets in Fig 6 and human DEGs.**

| **Gene_set** | **Overlap_upregulation** | **Overlap_downregulation** |
| --- | --- | --- |
| GSE9650_EFFECTOR_VS_EXHAUSTED_CD8_TCELL_DN | TNFRSF9, CXCL13, ADGRG1, VCAM1, CADM1, HLA-DMA | SSBP2, DDIT4, DUSP6, TM4SF1, SPP1, FHL1 |
| GSE9650_NAIVE_VS_EXHAUSTED_CD8_TCELL_DN | CCR5, CTLA4, TNFRSF9, IKZF4, ETV1 | HIST1H2BD, STX3, DUSP8, AHNAK, TM4SF1, PERP, FHL1 |
| GSE9650_EFFECTOR_VS_MEMORY_CD8_TCELL_UP | PDCD1, TNFRSF9, ENTPD1, CDCA3 | CRIP2, PERP, ANXA1, CX3CR1, IL12RB2, GZMK |
| GSE9650_EXHAUSTED_VS_MEMORY_CD8_TCELL_UP | ETV1, HLA-DMA, ADGRG1, ENTPD1 | DDIT4, TM4SF1, PERP, MS4A1, LGMN, CCR6 |
